# Economic burden of dengue in Puerto Rico, 2010–2023

**DOI:** 10.1186/s40249-026-01412-1

**Published:** 2026-01-27

**Authors:** Daniel Camprubí-Ferrer, Maile B. Thayer, Zachary J. Madewell, J. Mac McCullough, Liliana Sánchez-González, Aidsa Rivera, Janice Perez-Padilla, Dania M. Rodriguez, Jomil Torres Aponte, Michael A. Johansson, Gabriela Paz-Bailey, Vanessa Rivera-Amill, Melissa Marzan-Rodriguez, Laura E. Adams

**Affiliations:** 1 Barcelona Institute for Global Health (ISGlobal), Barcelona, Spain; 2 International Health Department, Hospital Clínic Barcelona, Barcelona, Spain; 3 Faculty of Medicine and Health Sciences, University of Barcelona (UB), Barcelona, Spain; 4 Division of Vector-Borne Diseases, Centers for Disease Control and Prevention, San Juan, Puerto Rico; 5 Boise State University, Boise, ID USA; 6 Division of Epidemiology & Research, Puerto Rico Department of Health, San Juan, Puerto Rico; 7 Ponce Health Sciences University, Ponce Research Institute, Ponce, Puerto Rico

**Keywords:** Dengue virus, Cost of illness, Surveillance system, Arboviral diseases, Underreporting adjustment, Multiplier model, Economic evaluation, Puerto Rico, Caribbean

## Abstract

**Background:**

Dengue remains a major public health challenge, particularly in endemic areas like Puerto Rico, where its economic burden is substantial. This study aimed to update the economic burden of dengue in Puerto Rico using recent data from patients, hospitals, and insurance companies, providing a clearer picture of the current situation. We estimated the total number of dengue cases with fever who sought care by adjusting for underreporting through a robust statistical framework linking island-wide passive surveillance data to sentinel acute febrile illness surveillance.

**Methods:**

We obtained cost data from hospitals and conducted interviews with a random sample of people diagnosed with dengue (*n* = 101) from December 2021–November 2022, collecting detailed information on direct medical costs, non-medical costs, and indirect costs. We analyzed median, epidemic and long-term dengue incidence patterns from 2010–2023. We conducted a cost-of-illness analysis using Bayesian multiplier methods to adjust for underreporting, followed by a bottom-up costing approach during a typical median incidence year and an epidemic year to illustrate the current economic burden of dengue in Puerto Rico.

**Results:**

In the median incidence year (2014), from 597 reported dengue cases we estimated 4500 [95% credible interval (95% CrI): 3700–5400] outpatient and 3900 (95% CrI: 3200–4700) hospitalized cases. During an epidemic year (2010), these figures rose substantially from the reported 10,359 dengue cases to an estimated 77,300 (95% CrI: 64,600–93,200) outpatient and 67,300 (95% CrI: 56,100–81,700) hospitalized cases. The median cost per hospitalized dengue case was 5200 USD for children and USD 6800 for adults, while outpatient costs were 2300 USD for children and 2700 USD for adults. Direct medical costs and indirect costs constituted the largest share of total costs. The total economic burden was 1.1 billion USD (95% CrI: 785 million–1.6 billion) during the epidemic year, compared to 62.7 million USD (95% CrI: 45.9–95.4 million) in the median incidence year.

**Conclusions:**

These findings highlight the considerable financial strain of dengue, particularly during epidemics, and underscore the urgent need for enhanced resource allocation, effective prevention strategies, and policy interventions to mitigate the economic impact of future outbreaks.

**Graphical Abstract:**

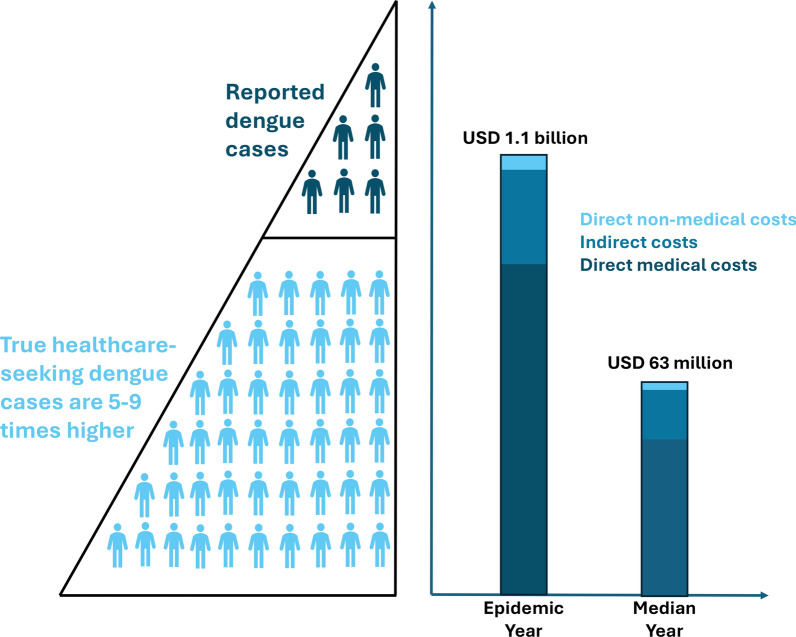

**Supplementary Information:**

The online version contains supplementary material available at 10.1186/s40249-026-01412-1.

## Background

Dengue, a mosquito-borne acute febrile illness caused by four serotypes, is the most prevalent arboviral disease globally [1–3]. With ongoing transmission reported in 128 countries, dengue virus (DENV) infections threaten over five billion people, resulting in global estimates as high as 390 million infections and 96 million symptomatic cases in epidemic years and 105 million infections and 51 million symptomatic cases in a typical year, primarily affecting children, adolescents and young adults [1, 4–8]. It is estimated that 75–85% of all DENV infections are asymptomatic [9]. Symptomatic cases typically present with fever, myalgia, retro-orbital pain, rash, headache, nausea and vomiting [10–12]. Patients can progress to severe disease due to severe plasma leakage, and present with shock, respiratory distress, hemorrhage, and organ impairment [13]. These symptoms incur both direct costs to healthcare systems and patients and indirect costs due to missed work or school days [14].

Dengue is endemic in Puerto Rico where large epidemics historically occur every 3–5 years [15, 16]. From 2010 to 2020, the island reported 20,675 confirmed cases and 9187 probable cases [16–18]. Dengue incidence was low following the Zika outbreak in 2016, with few cases reported from 2017 to 2019 likely due to short-term cross-protective immunity. During 2020–2023, case numbers returned to low-endemic levels. In 2024, a dengue epidemic was declared in Puerto Rico [19–21]. This evolving dengue landscape in Puerto Rico necessitates an updated assessment of the economic costs associated with the disease.

A previous analysis conducted from 2002 to 2010 estimated the average aggregate annual costs of treated dengue cases in Puerto Rico to be 38.7 million U.S. dollars (USD) (10.40 per capita, in 2010 USD) [22]. Including costs from dengue surveillance and vector control activities, the economic burden increased to 46.4 million USD (12.47 per capita). This burden represented 0.06% of Puerto Rico’s Gross Domestic Product (GDP) in 2010 [22], and exceeded the 0.03% GDP per capita burden estimated for 12 Southeast Asian countries [23]. Furthermore, dengue episodes resulted in an average of 30.5 days of lost productivity among patients and caregivers per case, including 7.2 days of absenteeism from work or school with each episode [22]. These cost estimates relied on an underreporting factor calculated via a capture-recapture study of two surveillance systems from 1991 to 1995 [22, 24].

Numerous studies have estimated the economic burden of dengue in other endemic regions, including Colombia, Vietnam, Mexico, and India, highlighting both the direct medical costs and broader societal impacts of the disease [25–28]. These studies emphasize the disproportionate burden on low-income populations, the high costs of hospitalization, and the potential for vaccination and vector control to reduce economic impact. However, dengue burden estimates remain highly context-specific due to differences in healthcare systems, surveillance, and disease severity. In Puerto Rico, the health system is similar to the U.S. model, although with some limitations in federal funding provided to the territory [29]. Most residents are covered by either the public government health insurance program or private insurance, and care for dengue patients is primarily provided through emergency departments, hospitals, and outpatient clinics. Laboratory confirmation for dengue is typically conducted by the Puerto Rico Department of Health (PRDH) or the U.S. Centers for Disease Control (CDC) Dengue Branch. This structure influences both the accessibility of dengue care and the reporting of cases and is important for interpreting the economic burden estimates presented in this study.

This study aimed to provide an updated and comprehensive evaluation of the current economic burden of dengue illness in Puerto Rico, including healthcare system costs, patients’ out-of-pocket expenses, social costs, and lost productivity. To achieve this, we incorporated updated data from 2021–2022 dengue cases, refined underreporting estimates and evaluated both direct and indirect costs. Beyond illness-related costs, dengue also imposes economic burden through activities aimed at disease prevention and control, such as epidemiological surveillance, laboratory testing to confirm cases, and vector control programs targeting mosquito populations. By including the annual costs of these activities alongside the costs of illness, hospitalization, healthcare system expenditures, patients’ out-of-pocket expenses, and lost productivity, this study provides a comprehensive estimate of the total economic impact of dengue in Puerto Rico. To summarize the economic burden under variable epidemiological conditions, we characterized costs for both a median incidence year and an epidemic year. Accurately quantifying the full extent of the dengue burden is important for policymakers, as it enables efficient allocation of resources and helps mitigate the economic impact of the disease.

## Methods

### Study design and data sources

In this study, we assessed the costs of dengue in Puerto Rico from a societal perspective, with cost components disaggregated to allow for potential estimation from alternative perspectives. All costs were adjusted to 2023 USD using the Consumer Price Index published by the U.S. Bureau of Labor Statistics [30]. We estimated direct and indirect costs of dengue illness in Puerto Rico from hospitalized, outpatient, and fatal cases. Confirmed and probable dengue cases were identified from two sources, Puerto Rico’s Passive Arboviral Disease Surveillance System (PADSS) and the Sentinel Enhanced Dengue Surveillance System (SEDSS). PADSS is the arboviral surveillance database for PRDH [31]. Healthcare providers report suspected dengue cases to PADSS based on clinical suspicion, with confirmation through laboratory testing conducted by PRDH. Dengue cases are defined as laboratory-confirmed if positive by reverse transcription-polymerase chain reaction (RT-PCR) testing for specimens collected within 7 days of symptom onset [32]. Probable dengue cases are defined as those positive for Immunoglobulin M (IgM) antibody capture enzyme-linked immunosorbent assay (ELISA) for anti-DENV antibodies for specimens collected > 3 days after symptom onset [18]. We obtained dengue case data reported to PADSS from 2010–2023 [33].

SEDSS is a facility-based active surveillance system managed by the Ponce Health Sciences University and the CDC Dengue Branch that actively monitors acute febrile and respiratory illnesses within emergency departments across Puerto Rico [18, 34–36]. SEDSS currently operates at three sites: Auxilio Mutuo Hospital (AM), a tertiary care facility in the San Juan Metro Area; Centro Médico Episcopal San Lucas (CMESL) in Ponce, a tertiary acute care facility; and Centro de Emergencia y Medicina Integrada (CEMI), an outpatient acute care clinic in Ponce. In SEDSS, patients are eligible for enrollment if they report fever upon presentation or within the past week (oral temperature ≥ 38 °C, axillary temperature ≥ 38.5 °C), or cough/dyspnea with or without fever (since the beginning of COVID-19 pandemic) within the last 14 days. All patients have DENV RT-PCR testing or IgM testing performed based on symptom onset date and the criteria defined above [18, 32].

### Costs study population and sampling

Dengue cases with positive results by RT-PCR or IgM from both surveillance systems were ordered by date, prioritizing the most recent cases to minimize recall bias [13]. Although no formal sample size was calculated, consistent with prior literature, we aimed to assess at least 100 dengue cases diagnosed after January 2020 [22]. Eligible patients, or their legal representatives, were invited to participate through phone calls, regardless of age or clinical outcome. Verbal informed consent was obtained from all participants or their legal surrogates. Residents outside Puerto Rico and individuals unable to provide verbal informed consent were excluded from the study. Participants who tested positive for dengue by RT-PCR and presented with fever [following 2015 Council of State and Territorial Epidemiologists (CSTE) clinical criteria of dengue-like illness] were classified as confirmed dengue cases. Participants meeting the 2015 CSTE clinical criteria of dengue-like illness and testing positive for anti-DENV IgM ELISA were classified as probable dengue cases. Participants not meeting the 2015 CSTE clinical criteria for confirmed or probable dengue were excluded from the study [33].

### Data collection and estimation of costs

After obtaining informed consent, interviewers followed a structured questionnaire to collect information about the patient’s duration of illness, use of medical services, the impact of dengue on schooling, work productivity and leisure time, out-of-pocket illness-related payments and income loss, as well as the time and income loss of caregivers due to the patient’s illness (Supplementary Methods 1). The questionnaire was developed based on previous cost-of-illness studies and reviewed by subject matter experts. Interviewers received training and followed a predefined protocol, and completed questionnaires were reviewed for completeness and internal consistency. Interviews were conducted by phone between July and October 2022, with data entered into the Research Electronic Data Capture (REDCap) system (REDCap consortium; version 5.20.11). We classified patients according to highest acuity level of care received—fatal case, inpatient hospitalization, or outpatient care.

Information regarding direct medical costs (DMC), direct non-medical costs (DNMC), and indirect costs was collected from multiple sources. Out-of-pocket non-hospital DMC and DNMC, as well as indirect costs were collected through structured interviews with patients or legal surrogates. Hospital DMC data were obtained from the financial departments of the six most frequently visited hospitals in our sample, representing 64.4% (58/90) of hospital admissions among our participants. These DMC data included detailed costs incurred during hospital admission, such as pharmacy, laboratory, radiology, emergency department services, medical and surgical supplies, and inpatient care [general wards, pediatrics, intensive care units (ICU)]. DNMC included meals, transportation to healthcare facilities and parking, among others.

To estimate the costs associated with dengue cases in Puerto Rico, we supplemented the hospital-sourced DMC data with average cost estimates provided by Puerto Rico government health insurance plans and private insurance companies, which reflect the amounts paid by insurers for each dengue case. These averages were stratified by age group (children < 18 years, adults ≥ 18 years) and were used to estimate the costs for dengue cases where hospital data were unavailable. PRDH estimates included total DMC for patients with the primary diagnostic code A90 “Dengue fever (classic dengue)” or A91 “Dengue hemorrhagic fever” from the International Classification of Diseases, 10th Edition (ICD-10). Additionally, costs associated with dengue diagnostic testing were estimated based on tests performed on suspected dengue cases submitted to PADSS from 2020–2022, covering expenses such as reagents, sample transport, and laboratory personnel time. These cost estimates were derived from known costs at the CDC Dengue Branch reference laboratory. For patients with missing cost information from any source, imputation was done using the average cost for each category based on age (children vs. adults) and the highest level of healthcare received. Table 1 provides a detailed list of DMC and DNMC included in the study.

**Table 1:** Detailed classification of direct medical and direct non-medical costs

Direct medical costs (DMC)	Days of hospital admission in pediatrics / general ward Days of admission to an ICU Ambulance trip Hospital consultation fees Dengue diagnostic tests Laboratory tests during hospital admission Medicines during hospital admission Radiology and ultrasound Medical and surgical devices Consultation to specialists during hospital admission Other DMC during hospital admission Consultation at the emergency room Visits to private clinics or medical offices Outpatient consultation fees after hospital admission Laboratory tests after hospital admission Medicines after hospital admission Transportation of samples to reference laboratories Work time of laboratory personnel at reference laboratories for the diagnosis of dengue
Direct non-medical costs (DNMC)	Meals Transportation to health facilities Parking Other DNMC
Indirect costs	Patient’s work absenteeism (outpatient and hospitalized cases) Patient’s school absenteeism (outpatient and hospitalized cases) Caregivers’ absenteeism (outpatient and hospitalized cases) Value of statistical life lost (fatal cases)

*ICU* intensive care unit

Indirect costs were estimated based on the number of school days lost for children and work absenteeism days for adults, as reported in patient interviews. Following the methodology of Halasa et al*.*, we estimated indirect costs of 51.30 USD per day of schooling lost per dengue episode in children [22]. This figure was calculated by dividing the most recent annual budget of the Puerto Rico Department of Education by the total enrollment in primary and secondary schools, and then dividing by the number of school days in a year [22, 37, 38]. While this does not represent productivity loss in the adult labor market, it reflects the educational investment lost per day of missed school and serves as a proxy for the societal cost of disrupted schooling. The economic cost per day of work absenteeism for adult patients and working-age children with a job was determined using Puerto Rico’s minimum daily wage of 70.04 USD [39]. The economic cost for days of care provided by caregivers was estimated using the same minimum daily wage.

### Estimation of annual healthcare-seeking dengue cases

To project the annual number of patients with acute febrile dengue seeking care, we compared population incidence data reported in PADSS to population incidence in SEDSS. In practice, dengue cases identified through SEDSS are also reported in PADSS; however, in this analysis, we differentiate between the two surveillance systems. Here, we define non-SEDSS PADSS cases (PADSS^NS^) as cases that were reported in PADSS but not captured through SEDSS. By leveraging the relationship between PADSS^NS^ and SEDSS, we calculate overall adjustment factors that account for underreporting. By basing these multipliers on PADSS^NS^ rather than all PADSS cases, we ensure that resulting adjustment factors can be applied to the entire island, including areas not covered by SEDSS, to estimate the total dengue health-seeking burden across Puerto Rico. These adjustment estimates were calculated separately for each of the three SEDSS sites and their respective municipalities and then synthesized into one island-wide estimate. We estimated these numbers separately by age group, and overall for hospitalized (using data from AM and CMESL only) and outpatient (using data from all three sites) cases.

We developed a statistical framework to estimate the ratio of total acute febrile dengue cases receiving hospitalized or outpatient care relative to numbers of probable and confirmed cases reported in PADSS^NS^. For one reported PADSS^NS^ case, the overall adjustment factor can be represented by the ratio of SEDSS case incidence (adjusted for underreporting in SEDSS) to PADSS^NS^ case incidence (adjusted for underreporting in PADSS^NS^).

Since we cannot directly observe the number of individuals with dengue who seek care, we assume that healthcare-seeking behavior for individuals with symptomatic dengue is similar to that of individuals with acute febrile illness. To be reported in SEDSS (${p}_{report.SEDSS}$), a person must seek care (${p}_{present}$), enroll (${p}_{enroll}$), and test positive ($sensitivity$) for dengue at a SEDSS facility. The probability of attending SEDSS (${p}_{attend.SEDSS}$) then represents the proportion of people with acute febrile illness who attended (sought care and enrolled; ${p}_{present}*{p}_{enroll}$) a SEDSS facility within the catchment area.

**Fig. 1:**
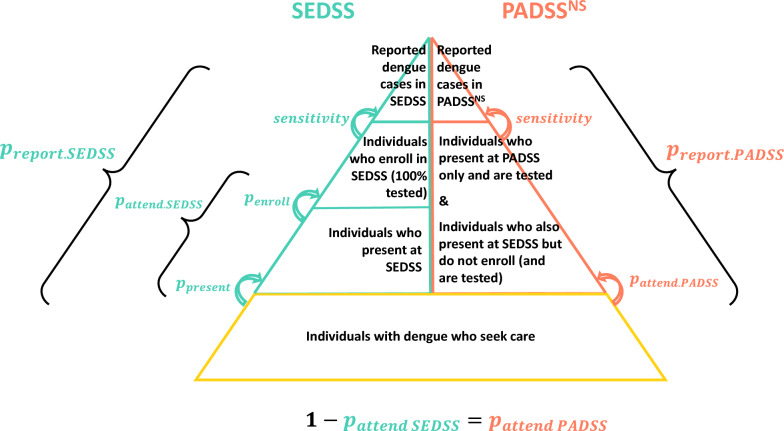
Dengue underreporting pyramid for the Sentinel Enhanced Surveillance System (SEDSS) and Passive Arboviral Surveillance System (PADSS) in Puerto Rico. The pyramid illustrates the different steps in the process for being reported in either surveillance system within a SEDSS catchment area. Symptomatic individuals with dengue seek care at either SEDSS or non-SEDSS PADSS (PADSS^NS^) sites. To be reported in SEDSS (${p}_{report.SEDSS}$), individuals must seek care (${p}_{present}$), enroll (${p}_{enroll}$), and then test positive ($sensitivity$) for dengue at a SEDSS facility. The probability of attending a SEDSS facility can be represented as the product of the probability of presenting and enrolling at that SEDSS site (${{p}_{attend.SEDSS}=p}_{present}*{p}_{enroll}$). To be reported in PADSS^NS^, individuals must attend a PADSS facility (${p}_{attend.PADSS}$) and then test positive for dengue ($sensitivity$). Within this catchment area, individuals who are seeking care but do not attend a SEDSS facility (i.e., they either present at SEDSS but then do not enroll, or they present at a PADSS facility) are instead noted as attending a PADSS facility (${p}_{attend.PADSS}=1-{p}_{attend.SEDSS}$). In both surveillance systems, individuals are reported as probable or confirmed dengue cases

Analogously, to be reported in PADSS^NS^ (${p}_{report.PADSS}$), a person must attend a PADSS facility (${p}_{attend.PADSS}$) and test positive ($sensitivity$) for dengue at that PADSS facility. Anyone who is seeking care for acute febrile illness and does not attend a SEDSS facility instead attends a PADSS facility. In other words, the probability of attending PADSS, ${p}_{attend.PADSS}$, can be calculated as the complement of ${p}_{attend.SEDSS}$ (${p}_{attend.PADSS}=1-{p}_{attend.SEDSS};$ Fig. 1).

From these estimates, we derived the ratio of dengue incidence from SEDSS compared to PADSS^NS^ to estimate the number of total febrile dengue patients seeking care for every probable and confirmed dengue case reported in PADSS^NS^. We estimated underreporting factors for adults, children, outpatient, hospitalized, and fatal cases.

For the underreporting adjustment for fatal cases, we employed a simpler approach. We used numbers from a previous study conducted in Puerto Rico from 2010 to 2012, as it remains the most comprehensive and setting-specific source on fatal dengue-like acute febrile illnesses, with complete testing and direct comparison to the surveillance system still in use [18]. The methodology, surveillance infrastructure, and underreporting have likely remained consistent over time. From this study, we used the total number of fatal cases reported in PADSS out of the total number of laboratory-confirmed dengue fatal cases to calculate the multiplier.

The models were fitted in Stan [40], and convergence was assessed using the Gelman-Rubin diagnostic [41]. Detailed statistical methods and model fitting procedures are provided in Supplementary Methods 2 and Table S1.

### Cost analysis and sensitivity analysis

We estimated total costs, as well as DMC, DNMC, and indirect costs per dengue case, by age group, highest level of healthcare received (hospitalized and outpatient cases), and fatal cases. Unit costs for outpatient, hospitalized, and fatal cases were estimated separately and then applied to the adjusted number of cases for each category, ensuring that aggregate costs reflect the epidemiological distribution of case severities rather than the composition of the cost sample. Overall costs were computed using the weighted average cost of pediatric and adult patients using adjusted case counts. All costs were standardized to 2023 USD to align with the last year of data used in the analyses.

For each cost parameter, we drew 10,000 independent samples from a log-normal distribution using the mean and standard deviation from our dataset, ensuring that those metrics were preserved in the samples. To comprehensively summarize cost distributions, we present both mean [and standard deviation (SD)] and median [and 95% credible interval (95% CrI)] estimates. The mean and SD are traditionally used in economic evaluations; however, they are sensitive to outliers and skewed data such as cost and disease distributions. As a result, we also present the median and 95% CrI. Statistical analyses were performed using R software, version 4.1.1 (R Foundation for Statistical Computing, Vienna, Austria) [42].

We analyzed dengue incidence data from 2010 to 2023, focusing on three representative time periods: an epidemic year (2010), a median incidence year (2014), and an eight-year total (2010–2014, 2021–2023). Together, these timeframes capture the range of typical dengue transmission dynamics and long-term trends in Puerto Rico. The period from 2015 to 2020 exhibited atypically low dengue incidence in Puerto Rico, likely due to disruptions from the Zika epidemic, which temporarily altered dengue immunity, and COVID-19-related movement restrictions. We also generated an eight-year total estimate to reflect long-term patterns and provide a practical reference point for expected long-term disease and costs to inform dengue prevention, surveillance, and control strategies. Together, these three representative time periods allow us to assess the costs of dengue in Puerto Rico under varying epidemiological conditions.

We estimated the aggregate cost of fatal cases as the value of statistical life (VSL) lost in addition to DMC, DNMC, and pre-death indirect costs of hospitalized dengue cases. The VSL corresponds to how much money society would be willing to spend to reduce the chances of dying across a population. In the absence of a federal agency Puerto Rico-specific VSL estimate, we applied the U.S. VSL in the main analysis, consistent with federal economic evaluation practices. The VSL, a standard measure in public health assessments, such as those for environmental regulation assessment and vaccine valuation [43, 44], was calculated using the U.S. Environmental Protection Agency’s estimation of 9.27 million USD per life lost [45, 46].

We conducted several sensitivity analyses to assess the robustness of our findings under varying assumptions. We assessed five scenarios to evaluate our assumptions regarding missing cost data and the estimation of costs for fatal cases. For scenario A, we used 0 USD for missing answers, assuming participants who did not report specific cost information incurred no expenses. For scenario B, we estimated costs for fatal cases using information on length of stay and admission to the ICU from a previous report on dengue deaths in Puerto Rico [18]. For scenario C, we adopted the approach of Halasa et al. for estimating costs of fatal cases [22]. Different from our main approach with the VSL which estimates society’s willingness to pay for reductions in the likelihood of death, Halasa et al*.* values loss of life in terms of lost economic productivity (the product of years of life lost from dengue and median income). For scenario D, given income differences between Puerto Rico and the continental U.S., we used VSL estimates imputed for Puerto Rico from a hedonic wage method extrapolated from Chile (6.8 million in 2023 USD) [47]. In Scenario E, we use the less conservative VSL estimate produced by the U.S. Department of Transportation (13.2 million in 2023 USD) [48].

In the absence of dengue-specific healthcare-seeking behavior, we assumed in the main analysis that the probability of presenting at a SEDSS facility for dengue was equivalent to that for a fever (${p}_{present}$). To assess this assumption, we conducted sensitivity analyses varying the assumed care-seeking probability (${p}_{present}$) by ± 10% and ± 20%.

Lastly, we estimated the cost per capita across all categories for a median incidence year (2014) and an epidemic year (2010) by calculating the aggregate costs over Puerto Rico’s population during those years, which were 3,534,874 and 3,721,525, respectively [49].

### Surveillance and vector control activities

To estimate the aggregate annual economic cost of dengue in Puerto Rico, we incorporated the costs of dengue vector control activities in addition to epidemiological and laboratory testing surveillance. Vector control activities included personnel costs, travel, laboratory supplies (entomological and molecular), field (including maintenance supplies), mobile laboratory (including maintenance), storage, insurance, and others.

Although our primary analyses stratified costs by a median incidence year and an epidemic year, these years do not fully represent current spending on surveillance and vector control. Specifically, before 2016, there were no dedicated US CDC funds for vector control, and we lacked detailed cost data for vector control activities. Additionally, surveillance systems evolved from passive surveillance in 2010 to include enhanced sentinel surveillance sites by 2014. To address these limitations, we incorporated surveillance and vector control costs from a more recent year, 2023, to provide updated cost estimates for vector control activities. Our intention was therefore to estimate the current economic costs that typical (median incidence) dengue dynamics would impose under today’s surveillance and vector control structure, rather than reproduce historical spending levels.

## Results

### Participant characteristics

Between December 2021–November 2022, 175 potential dengue cases were identified and included in the list of participants to be contacted by interviewers. Five participants were excluded based on clinical criteria for probable or confirmed dengue, 39 did not answer the phone calls, 17 declined to participate, and 13 did not complete the interview, resulting in 101 cases included in the study (recruitment rate: 59.4%).

Among 101 participants, 59 (58.4%) were children, 98 (98.0%) had health insurance (54.0% public, 44.0% private) (Table S2), and 90 (89.1%) required hospital admission, with an average length of stay of 5.5 days (range: 1–14 days). Most (97; 96.0%) had a positive dengue result by RT-PCR; the remaining four had positive results for dengue by IgM. Only five (5.6%) hospitalized cases required ICU admission, with an average stay of 3.6 days (range: 1–8 days). The median number of days of schooling missed and/or work absenteeism due to dengue were 15 days for hospitalized children and adults, whereas ambulatory children and adults missed 3 and 9 days, respectively (Table S3). For adults who reported both schooling and work absenteeism, the total number of days missed due to dengue was calculated as the sum of both types of absenteeism and monetized using the same per-day cost. Most patients (91; 90.1%) were assisted by caregivers for an average of 9 and 8 days in hospitalized children and adults; and 5 and 1 days in ambulatory children and adults, respectively.

### Underreporting of dengue cases

We estimated that 2% (95% CrI: 2–3%), 7% (95% CrI: 6–8%), and 2% (95% CrI: 2–3%) of residents with dengue seeking care presented and enrolled in SEDSS in the catchment areas of AM, CMESL, and CEMI, respectively (Table S4). Accounting for these proportions and comparing across sites, we estimated that overall, there were approximately 7.5 (95% CrI: 6.2–9.0) dengue outpatient cases and 6.6 (95% CrI: 5.5–8.0) dengue hospitalized cases for every probable and confirmed dengue case reported in PADSS^NS^ (Table 2). The relative incidence in SEDSS compared to PADSS^NS^ was generally more similar for children compared to adults (Table S4), resulting in overall lower adjustment factors for children than for adults. For children, we estimated that there were approximately 2.9 (95% CrI: 2.2–3.8) dengue outpatient cases and 2.9 (95% CrI: 2.2–3.8) dengue hospitalized cases for every probable and confirmed case reported in PADSS^NS^. For adults, we estimated that there were approximately 10.7 (95% CrI: 8.3–13.9) dengue outpatient cases and 7.1 (95% CrI: 5.4–9.4) dengue hospitalized cases for every probable and confirmed case reported in PADSS^NS^.

**Table 2:** Estimated adjustment factors

	Outpatient	Hospitalized	Fatal
Overall	7.5 (6.2–9.0)	6.6 (5.5–8.0)	1.1 (1.1–1.3)
Children	2.9 (2.2–3.8)	2.9 (2.2–3.8)	1.2 (1.0–2.2)
Adults	10.7 (8.3–13.9)	7.1 (5.4–9.4)	1.1 (1.0–1.3)

The multiplier ratios for one [non-Sentinel Enhanced Dengue Surveillance System (SEDSS)] Passive Arboviral Disease Surveillance System (PADSS) reported probable or confirmed dengue case are presented as median estimates (95% credible intervals) separately for hospitalized, outpatient and fatal cases and age categories (overall, children < 18 years, and adults ≥ 18 years). The multipliers for hospitalized and outpatient cases are applied to reported non-fatal (non-SEDSS) PADSS cases, and the fatal case multipliers are applied to reported fatal (non-SEDSS) PADSS dengue cases

Accounting for underreporting of fatal cases, there were approximately 1.1 (95% CrI: 1.1–1.3) true fatal cases for every reported fatal case in PADSS. These numbers were similar for children and adults with adjustment multipliers of 1.2 (95% CrI: 1.0–2.2) and 1.1 (95% CrI: 1.0–1.3), respectively.

### Estimated dengue burden in Puerto Rico

From 2010 to 2023, 32,417 dengue cases (confirmed: 22,718; probable: 9699) were identified through PADSS^NS^, of which 17,664 (54.5%) were adults and 14,374 (44.3%) were children. Of those reported dengue cases, 86 were reported fatal cases, of which the majority (92%) were adults. Annual reported cases ranged from 20 in 2017 to 10,359 in 2010. The median year for reported incidence was 2014, with 597 cases (Table 3, Fig. S1).

**Table 3:** Reported and estimated outpatient, hospitalized, and fatal dengue cases for a median incidence year, an epidemic year, and a long-term total by age group, Puerto Rico

		Reported cases (non-SEDSS PADSS)	Estimated total outpatient cases, Median (95% CrI)	Estimated total hospitalized cases, Median (95% CrI)	Reported fatal cases (non-SEDSS PADSS)	Estimated total fatal cases, Median (95% CrI)
Median incidence year (2014)	All	597	4500 (3700–5400)	3,900 (3200–4700)	2	2 (2–3)
	Children	201	600 (400–800)	600 (400–800)	1	1 (1–2)
	Adults	389	4100 (3200–5400)	2,800 (2100–3600)	1	1 (1–1)
Epidemic year (2010)	All	10,359	77,300 (64,600–93,200)	67,300 (56,100–81,700)	38	43 (40–49)
	Children	4831	13,900 (10,600–18,200)	14,100 (10,800–18,500)	4	5 (4–9)
	Adults	5400	57,600 (44,400–74,700)	38,400 (29,200–50,500)	34	38 (36–43)
8-year total (2010–2014, 2021–2023)	All	31,195	232,700 (194,500–280,700)	202,800 (169,000–245,900)	86	97 (90–110)
	Children	13,833	39,800 (30,400–52,100)	40,300 (30,800–52,900)	7	8 (7–15)
	Adults	16,987	181,200 (139,600–235,100)	120,900 (91,800–158,700)	79	89 (83–101)

Reported cases are probable and confirmed dengue numbers from (non-SEDSS) PADSS. Incidence and case numbers were estimated using 2014 (median) and 2010 (epidemic) data, and case multipliers were calculated using 2010–2023 data. The eight-year total represents long-term trends in Puerto Rico. The eight years in the long-term total include all years in the 2010–2023 except for 2015–2020, which was marked by atypical dengue incidence in Puerto Rico likely due to the Zika epidemic and COVID-19 pandemic. The total numbers are larger than the sum of the numbers for children and adults due to missing values for age. *SEDSS* Sentinel Enhanced Dengue Surveillance System; *PADSS* Passive Arboviral Disease Surveillance System; *CrI* Credible interval

In the median incidence year, 2014, there were an estimated 4500 (95% CrI: 3700–5400) outpatient cases, 3900 (95% CrI: 3200–4700) hospitalized cases, and 2 (95% CrI: 2–3) fatal cases due to dengue after adjusting for underreporting. In an epidemic year, 2010, the estimated total cases were approximately 17 times higher with 77,300 (95% CrI: 64,600–93,200) outpatient cases and 67,300 hospitalized cases (95% CrI: 56,100–81,700), 43 (95% CrI: 40–49) of which were fatal cases. In the long-term total, there were an estimated 232,700 (95% CrI: 194,500–280,700) outpatient cases, 202,800 (95% CrI: 169,000–245,900) hospitalized cases, and 97 (95% CrI: 90–110) fatal cases due to dengue over the eight-year timeframe.

### Economic burden of dengue

The estimated total and disaggregated costs (DMC, DNMC, and indirect costs) per dengue case, stratified by age group and hospitalization status, are detailed in Table 4. For hospitalized cases (weighted average of adults and children), DMC comprised the largest cost component, representing 70.6% of average total costs, followed by indirect costs (25.1%), and DNMC (4.2%). Similarly, for outpatients (weighted average of adults and children), DMC were the major cost component (69.8%), followed by indirect costs (27.2%), and DNMC (3.0%). The relatively high DMC of outpatient cases were primarily driven by emergency room and hospital visits that did not require overnight stays and laboratory procedures covered by insurance or processed through central referral laboratories.

**Table 4:** Average and median cost per dengue case by care setting, type of cost, and age group, 2010–2023, Puerto Rico

Setting and type of cost	Children		Adults		Weighted average	
	Mean (SD)	Median (95% CrI)	Mean (SD)	Median (95% CrI)	Mean (SD)	Median (95% CrI)
Hospitalized (total)	**5700** **(2300)**	**5200** **(2700–11,500)**	**7500** **(3400)**	**6800** **(3100–15,900)**	**7100** **(3200)**	**6600** **(3600–13,400)**
Direct medical	4000 (2200)	3400 (1200–9600)	5300 (3200)	4600 (1600–13,200)	5000 (3000)	4400 (2000–10,900)
Direct non-medical	300 (300)	200 (100–1100)	300 (200)	200 (100–1000)	300 (300)	300 (100–800)
Indirect	1400 (600)	1300 (600–3000)	1900 (1200)	1600 (500–4800)	1800 (1100)	1600 (700–4000)
Outpatient (total)	**2400** **(900)**	**2300** **(1200–4600)**	**2800** **(400)**	**2700** **(2100–3800)**	**2700** **(500)**	**2700** **(2100–3600)**
Direct medical	1800 (800)	1700 (700–3900)	1900 (200)	1900 (1600–2300)	1900 (400)	1900 (1500–2400)
Direct non-medical	100 (150)	100 (0–500)	70 (60)	100 (0–200)	80 (80)	100 (0–200)
Indirect	500 (200)	500 (200–1100)	800 (400)	700 (300–1700)	700 (400)	700 (300–1500)
Fatal (total)	**9,275,700** **(2,446,300)**	**9,029,000** **(5,395,900–14,993,700)**	**9,277,500** **(2,446,300)**	**8,990,800** **(5,390,100–14,966,300)**	**9,277,300** **(2,446,300)**	**9,051,200** **(5,840,900–14,270,700)**
Direct medical	4000 (2200)	3400 (1200–9900)	5300 (3200)	4500 (1600–13,600)	5200 (3100)	4500 (1800–12,300)
Direct non-medical	300 (300)	200 (100–1100)	300 (200)	200 (100–900)	300 (300)	200 (100–800)
Indirect	9,271,400 (2,446,300)	9,025,300 (5,390,600–14,988,300)	9,271,900 (2,446,300)	8,985,600 (5,383,400–14,956,400)	9,271,800 (2,446,300)	9,045,800 (5,837,200–14,267,100)

*Direct medical costs* include hospital charges (e.g., emergency room, pharmacy, lab, radiology, inpatient care) or insurer reimbursements for outpatient care. *Direct non-medical costs* include patient and caregiver out-of-pocket expenses such as transportation, meals, and parking. *Indirect costs* reflect lost productivity due to missed school or workdays by patients and caregivers. For fatal cases, indirect costs are based on the value of a statistical life (VSL), using U.S. Environmental Protection Agency estimates (see Methods). *Weighted averages* reflect case distribution by age group within each setting. All results are presented in 2023 US dollars (USD). Both means with standard deviations and medians with 95% credible intervals are presented to provide a comprehensive summary of the results, as means are commonly used in economic analyses, while medians and credible intervals better reflect the skewed distribution of the data. Note that in some instances the median of the weighted average is larger than the median of the costs for children and adults due to the skewed distributions of costs and nonlinear nature of medians*. SD* standard deviation; *95% CrI* 95% credible interval

Hospital financing accounted for the majority of the cost estimations for hospitalized dengue patients (63.2% for children and 58.7% for adults), while insurance schedules contributed the most for outpatients (66.7% for children and 60.7% for adults) (Fig. S2). Out-of-pocket expenses collected from the detailed questionnaire had the second-highest contribution for both hospitalized and outpatient individuals, ranging from 29.1–40.0%.

For hospitalized cases, children incurred a lower median total cost per case (5200 USD, 95% CrI: 2700–11,500) compared to adults (6800 USD, 95% CrI: 3100–15,900) (Table 4). This difference was primarily driven by lower DMC for children (median 3400 USD, 95% CrI: 1200–9600) compared to adults (4600 USD, 95% CrI: 1600–13,200), followed by indirect costs, which account for the time spent by caregivers providing for patients (children: 1300 USD; adults: 1600 USD). Similarly, for outpatients, children had a lower median total cost per case (2300 USD, 95% CrI: 1200–4600) compared to adults (2700 USD, 95% CrI: 2100–3800). As with hospitalized cases, the main contributor to this difference was lower DMC for children (1700 USD, 95% CrI: 700–3900) compared to adults (1900 USD, 95% CrI: 1600–2300), followed by indirect costs (children: 500 USD, 95% CrI: 200–1100; adults: 700 USD, 95% CrI: 300–1700). The sensitivity analysis, assuming participants who did not report any specific cost information incurred no expenses, yielded slightly lower median costs of 6400 USD per hospitalized dengue case and 1400 USD per outpatient case (Table S5, Scenario A).

The estimated median cost per fatal dengue case was 9,051,200 USD (95% CrI: 5,840,900–14,270,700), with similar costs for adults and children (Table 4). These estimates were similar (median: 9,008,800 USD, 95% CrI: 5,847,200–14,043,600) when hospitalization and length of ICU admission data from Tomashek KM et al. was used for the estimation (Table S5, Scenario B) [18]. When estimating based on the economic value of years lost from premature death following the approach by Halasa et al*.*, cost estimations were substantially lower, at 638,200 USD (95% CrI: 365,900–664,600) for child and 550,100 USD (95% CrI: 546,900–559,200) for adult deaths (Table S5, Scenario C). Similarly, using a VSL hedonic wage method extrapolated to Puerto Rico yielded slightly lower estimates of overall costs for fatal cases, at 6,849,200 USD (95% CrI: 6,846,400–6,857,300) (Table S5, Scenario D). On the other hand, using the VSL estimate from the U.S. Department of Transportation yielded substantially higher overall costs for fatal cases, at 13,204,800 USD (95% CrI: 13,202,100–13,212,700) (Table S5, Scenario E).

### Aggregate annual cost of dengue illness

The aggregate annual cost of dengue illness in Puerto Rico, stratified by setting, cost type, and time period is summarized in Table 5 and Fig. S3. For the median incidence year, the total estimated cost fro dengue illness was 62.7 million USD (95% CrI: 49.5 – 95.4 million), translating to 18 USD per capita (95% CrI: 13 – 27). Hospitalized cases accounted for most of the costs (43.9%), followed by fatal cases (35.2%), and outpatient cases (20.9%). Indirect costs and DMC comprised the largest share (52.1% and 45.4%, respectively), with DNMC (2.5%) constituting the remaining portion. During an epidemic year, the aggregate cost soared to 1.1 billion USD (95% CrI: 785 million–1.6 billion), equivalent to 291 USD (95% CrI: 211–432) per capita. The cost distribution across settings and cost types in the epidemic year remained relatively consistent with the median year. Hospitalized cases again shouldered the largest burden (43.0%), followed by fatal cases (37.5%) and outpatient cases (19.5%). Indirect costs and DMC continued to be the dominant components (53.6% and 43.9%, respectively), followed by DNMC (2.4%).

**Table 5:** Aggregate annual cost (in thousands of 2023 US dollars, USD) of illness of projected dengue cases by setting and type of cost for a median incidence year, an epidemic year, and a long-term total in Puerto Rico

Year	Setting and type of cost	Hospitalized		Outpatient		Fatal		Total	
		Mean (SD)	Median (95% CrI)	Mean (SD)	Median (95% CrI)	Mean (SD)	Median (95% CrI)	Mean (SD)	Median (95% CrI)
Median incidence year	Total	**28,400** **(11,200)**	**26,100** **(13,500–56,600)**	**13,500** **(2300)**	**13,200** **(9900–18,700)**	**22,800** **(5300)**	**22,100** **(14,800–35,000)**	**64,700** **(12,800)**	**62,700** **(45,900–95,400)**
	Direct medical	20,000 (10,400)	17,600 (7300–46,700)	9300 (1300)	9200 (7100–12,300)	10 (5)	10 (5–24)	29,400 (10,600)	27,000 (16,100–56,300)
	Direct non–medical	1200 (800)	1000 (300–3400)	400 (300)	300 (100–1100)	< 1 (< 1)	< 1 (< 1–2)	1600 (900)	1400 (600–3800)
	Indirect	7100 (3900)	6200 (2500–16,900)	3700 (1600)	3400 (1600–7700)	22,800 (5300)	22,100 (14,700–35,000)	33,700 (6800)	32,900 (23,200–49,300)
Epidemic year	Total	**477,100** **(178,500)**	**441,900** **(236,900–915,600)**	**216,300** **(35,100)**	**212,400** **(159,100–297,000)**	**416,600** **(101,700)**	**404,500** **(254,500–646,600)**	**1,110,000** **(210,600)**	**1,083,400** **(784,500–1,607,400)**
	Direct medical	336,500 (165,400)	299,500 (129,500–757,500)	150,900 (21,900)	149,100 (113,700–198,900)	200 (100)	200 (100–600)	487,700 (168,700)	451,400 (270,700–915,100)
	Direct non–medical	20,500 (13,400)	17,100 (5500–56,000)	6600 (4700)	5400 (1700–18,700)	13 (10)	11 (3–40)	27,100 (14,300)	23,800 (9700–63,700)
	Indirect	120,100 (60,700)	106,200 (45,700–273,500)	58,800 (23,700)	54,300 (26,400–119,100)	416,300 (101,700)	404,300 (254,300–646,400)	595,200 (121,600)	581,100 (399,500–869,300)
8-year total (2010–2014, 2021–2023)	Total	**1,443,300** **(545,600)**	**1,334,500** **(709,600–2,787,300)**	**658,400** **(106,800)**	**646,800** **(485,000–903,600)**	**941,000** **(234,800)**	**913,200** **(568,000–1,475,600)**	**3,042,700** **(610,200)**	**2,952,500** **(2,124,600–4,509,200)**
	Direct medical	1,018,500 (505,800)	904,200 (388,900–2,309,000)	458,900 (65,500)	453,400 (347,200–602,100)	500 (300)	500 (200–1,300)	1,477,900 (515,300)	1,366,400 (817,600–2,783,500)
	Direct non–medical	61,600 (40,700)	51,100 (16,400–170,200)	20,000 (14,400)	16,400 (5000–56,700)	30 (20)	20 (7–100)	81,600 (43,300)	71,800 (29,000–193,400)
	Indirect	363,300 (186,000)	320,100 (136,000–832,400)	179,500 (72,900)	165,500 (80,500–363,700)	940,400 (234,800)	912,700 (567,700–1,475,300)	1,483,200 (310,600)	1,442,900 (991,700–2,179,900)

Both means with standard deviations and medians with 95% credible intervals are presented to provide a comprehensive summary of the results, as means are commonly used in economic analyses, while medians and credible intervals better reflect the skewed distribution of the data. The eight–year total represents long-term trends in Puerto Rico. The eight years in the long-term total include all years in the 2010–2023 period except for 2015–2020, which was marked by atypical dengue incidence in Puerto Rico likely due to the Zika epidemic and COVID-19 pandemic. *SD* standard deviation; *95% CrI* 95% credible interval

Varying the probability of seeking care at a SEDSS facility produced changes in aggregate costs of illness of approximate increases of 10–12%, and decreases of 12–17% in the parameter, depending on the variation in the parameter (Table S6). Overall results were only moderately sensitive to these changes, reflecting that the parameters used in the main analysis for presenting at a SEDSS facility are likely reasonable.

Factoring in the costs of human and vector surveillance and control activities in addition to the 62.7 million USD annual direct and indirect costs of dengue illness, the median annual aggregate cost of dengue in Puerto Rico during a median incidence year was estimated to be 66.7 million USD (95% CrI: 49.4–99.7 million) and represented 19 USD per capita (Table 6).

**Table 6:** Annual aggregate and per capita costs of dengue illness, surveillance and vector control in Puerto Rico for a median incidence year between 2010 and 2023

	Total costs (in millions of USD)		Costs per capita (in USD)		Percent
	Mean (SD)	Median (95% CrI)	Mean (SD)	Median (95% CrI)	
Illness costs for a median incidence year					
Direct	31.0 (10.7)	28.7 (17.4–58.2)	9 (3)	8 (5–16)	45%
Indirect	33.7 (6.8)	32.9 (23.2–49.3)	10 (2)	9 (7–14)	49%
Surveillance and vector control for 2023					
Human surveillance	2.2 (1.1)	2.0 (0.8–5.0)	1 (< 1)	1 (< 1–1)	3%
Vector surveillance and control	1.7 (0.9)	1.5 (0.6–3.9)	< 1 (< 1)	< 1 (< 1–1)	2%
Total	**68.6 (12.9)**	**66.7 (49.4–99.7)**	**19 (4)**	**19 (14–28)**	**100%**

Average and median aggregate and per capita costs with their standard deviation (SD) and 95% credible intervals (95% CrI) are shown for each type of cost (direct illness costs, indirect illness costs, human surveillance, and vector surveillance and control). Both means with standard deviations and medians with 95% credible intervals are presented to provide a comprehensive summary of the results, as means are commonly used in economic analyses, while medians and credible intervals better reflect the skewed distribution of the data. Per capita costs are calculated as costs per individual in the (2014) population. Their contribution (percent) to the total average annual economic costs are also shown in the last column. The year 2014 was used for a median incidence year for all illness costs (adjusted to 2023 USD), but surveillance and vector control costs were only available for one year (2023). As a result, costs are presented for a typical median incidence year only

## Discussion

Our findings confirm the substantial economic burden of dengue in Puerto Rico, driven primarily by DMC associated with hospitalization and treatment as well as indirect costs due to fatalities [7, 23]. This substantial financial strain affects individuals, families, and healthcare systems, as highlighted by previous research [25, 50]. This burden is particularly challenging for low- and middle-income countries, where dengue outbreaks can overwhelm hospitals and clinics, leading to resource shortages and compromised care for other health conditions [51]. Our study highlights this point further by demonstrating that the aggregate illness costs during an epidemic year soared to over 1 billion USD, exceeding 17 times the costs of a non-epidemic year. This dramatic increase emphasizes the critical importance of robust surveillance systems for early epidemic detection and timely implementation of public health interventions [21]. The exceptionally high number of dengue cases reported in 2010 was driven by a combination of widespread transmission of DENV-1, low pre-existing population immunity, and favorable environmental conditions for *Ae. aegypti* proliferation [19, 52]. These factors contributed to one of the largest recorded epidemics on the island, highlighting the extent to which epidemic years can dramatically escalate both disease burden and associated costs.

Our estimated annual cost of dengue illness (median 62.7 million; average 64.7 million USD for the median incidence year) is higher than the average estimate from another study from 2002 to 2010, 53.6 million (2023) USD [22]. Several factors contribute to this difference. First, while the average annual PADSS-reported cases from that study were more than three times as high as those in the median year analyzed here (1831 cases versus 597), the combination of our adjustment factors and updated costs yield higher overall estimates. Our underreporting method improves upon existing approaches by providing a comprehensive, context-specific framework that explicitly accounts for dengue severity, care-seeking and enrollment across multiple geographic areas, rather than relying on capture-recapture methods or predetermined factors. Our methods yielded higher adjustment factors for hospitalized cases but lower factors for ambulatory and fatal cases, which more fully capture variation in case detection. Furthermore, our updated individual costs are higher compared to those from Halasa et al. in every instance except for hospitalized adults. Our study incorporates more updated and comprehensive cost inputs with more recent and complete data on medical care practices, resource use, laboratory testing, and hospital and outpatient services. Our estimates for the 2010 epidemic further indicate how much higher costs can be during a major epidemic year. Additionally, the variation in dengue severity from year to year can substantially impact hospitalization rates and associated costs. Higher proportions of severe cases may occur during years when different serotypes or genotypes circulate, when population immunity is low, or when secondary infections are more common. Furthermore, changes in medical practices and treatment protocols for more severe cases over time could have introduced more expensive treatment options. Sector-specific inflation and advancements in medical technology also play a role in driving up healthcare costs over time. Unlike the previous study, which relied solely on passive surveillance, our analysis incorporated cases identified through enhanced surveillance methods, potentially providing a more accurate estimation of case numbers. Despite these differences, both studies underscore the substantial economic burden posed by hospitalized cases, which consistently represent the largest cost component due to the resource-intensive nature of severe disease management. This finding is critical in the context of dengue, given the strain that outbreaks place on healthcare systems.

The similarity in multipliers for outpatients and hospitalized patients may appear counterintuitive, as severe cases requiring hospitalization are generally more likely to be detected in the surveillance system. However, this similarity reflects several factors. First, hospitalized cases in passive surveillance systems like PADSS are predominantly identified based on clinical suspicion for dengue, which relies on presenting symptoms and testing capacity. Similarly, outpatients with severe symptoms are more likely to seek care in emergency settings, where surveillance systems like SEDSS operate actively. Second, outpatient dengue cases represent a diverse spectrum of severity, and milder cases may be captured in emergency rooms due to accessibility, particularly in resource-limited settings where primary care options are less prevalent. This could reduce the difference in detection rates between outpatient and hospitalized cases. Additionally, while hospitalized patients may seem more likely to be detected, certain barriers—such as delayed care-seeking or atypical presentations—may still lead to underreporting, particularly in non-pediatric populations. This fact is further reflected in the lower multiplier estimates for children for both hospital and outpatient settings. While children can be more susceptible to diseases, parents and caregivers tend to be more likely to seek care for their children regardless of the severity of disease, resulting in higher rates of healthcare seeking and lower rates of underreporting [53–57].

The reliance on emergency room-based surveillance may also introduce selection bias, as emergency care facilities typically serve patients with more acute or severe symptoms compared to those who visit primary care providers or pediatricians’ offices. This bias could result in a higher capture of severe outpatient cases in SEDSS and affect the relative multipliers for outpatients versus hospitalized cases. Further studies incorporating data from primary care and outpatient clinics would help refine estimates and better understand how care-seeking behavior influences underreporting.

Our results provide further evidence demonstrating that for every reported dengue case, there are 6–9 febrile outpatient and 5–8 hospitalized cases that go undetected. These ratios are different compared to earlier estimates, such as those from the 1990s using a single expansion factor of 2.4 for hospitalized and outpatient cases, and from Shankar et al., who estimated 5–9 for hospitalized cases and 21–115 for outpatients [22, 24, 58]. The 2.4 factor was derived from capture-recapture methods applied to hospitalizations for dengue during 1991–1995 [22, 24] and relied on data from a surveillance system that no longer exists. The later estimates share some similarities in methodology to the one used here, but the data were limited to a small rural subregion of Puerto Rico (Patillas and Guayama) and originated from an older surveillance system, and excluded all but two years of data to create the different multiplier estimates. Here, we improve upon these previous methods to integrate current surveillance systems, extrapolate across multiple regions of the island, and include as many years of relevant data as possible.

Recent studies further underscore the limitations of passive surveillance systems. For example, a placebo-controlled dengue vaccine efficacy study in Puerto Rico and Latin America found confirmed dengue incidence rates 10- to 25-fold higher in actively monitored cohorts compared to passive surveillance systems [59]. Such discrepancies highlight the challenges of relying solely on passive surveillance systems [34, 60, 61], which often fail to capture the complete scope of dengue incidence due to factors like healthcare-seeking behavior, diagnostic capacity, and awareness among healthcare providers. The implications for public health are profound: underestimation of dengue cases can result in suboptimal resource allocation, misinformed intervention strategies, and insufficient preparedness for outbreaks. Accurate estimation of the disease burden is crucial for effective planning and implementation of control measures, as seen in countries like Singapore, where enhanced surveillance has led to improved resource allocation and more effective outbreak management [62]. To improve upon previous methods, we developed a statistical framework that estimates unreported dengue cases by adjusting for clinical suspicion, testing practices, and patient catchments between surveillance systems. Our model offers a more refined approach to estimating true disease burden and highlights the ongoing challenges in dengue surveillance.

Children generally had lower total costs than adults, primarily due to lower DMC. Although children are often hospitalized at higher rates than adults, this may not always translate into higher costs, as the intensity or duration of treatment required for children may be less. However, we cannot definitively conclude this from our data, as factors such as length of stay and treatment intensity were not analyzed in this context [63]. Additionally, higher hospitalization rates in the elderly may also contribute to the higher costs observed in that age group [64]. The comparable indirect costs across age groups underscore the often-overlooked burden on caregivers, particularly in caring for sick children. This finding emphasizes the need for comprehensive support systems for families affected by dengue, including provisions for childcare and caregiver respite.

The substantial contribution of indirect costs to the overall economic burden highlights the broader societal impact of dengue. Productivity losses due to illness and caregiving responsibilities can have ripple effects on the economy, affecting sectors such as education, agriculture, and tourism [65]. For example, a study in Thailand and Brazil reported significant economic losses far surpassing the direct medical costs during dengue outbreaks due to decreased productivity, increased absenteeism, and lost tourism [66].

While the costs of surveillance and vector control activities represent a smaller fraction of the overall dengue burden compared to illness costs, these activities are crucial for effective disease prevention and control. Investing in robust surveillance systems and targeted vector control efforts can lead to earlier detection of outbreaks, improved case management, and ultimately, a reduction in the number of dengue cases and associated costs. Additionally, vector-control strategies may impact other mosquito-borne diseases such as Zika and chikungunya, increasing the potential benefit of these measures.

Environmental and seasonal conditions that support mosquito proliferation can substantially increase dengue transmission and associated costs, as illustrated by the 2010 epidemic. The potential for more frequent or intense outbreaks highlights the importance of sustained investment in early warning systems, targeted vector control, and preventive tools such as vaccination and improved housing infrastructure. Our findings suggest that interventions that reduce hospitalization rates—especially during epidemic periods—may yield substantial cost savings and should be prioritized in public health planning.

## Limitations

This study was subject to limitations. Medical care and medication costs can fluctuate over time, so results might not reflect costs over the full evaluation period. Selection bias is possible given the low response rate and limited information to compare respondents with nonrespondents. Additionally, the small sample size, skewed distribution of hospitalized and outpatient cases, and lack of de-aggregated costs from facility financial departments prevented us from evaluating how costs may vary by illness severity, location, insurance type, or year. The distribution of cases reflects data availability and healthcare-seeking patterns during the study period; however, to mitigate potential bias from this imbalance, we explicitly incorporated the observed case type variability into our analysis.

This study focuses on immediate costs associated with dengue episodes and does not capture potential long-term economic consequences (e.g., complications requiring additional care, future productivity losses, or impacts on educational attainment from illness-related absences). The study’s categorization of costs may also overlook potential impacts on tourism and businesses during outbreaks [65–67].

The study does not account for individuals with DENV who seek care without acute fever. Some of these individuals likely experience disease and indirect costs. However, fever is the most prevalent symptom of dengue, with acute febrile illness reported in 95% of probable and confirmed cases reported in (non-SEDSS) PADSS. We assume healthcare-seeking for symptomatic dengue mirrors that for acute febrile illness; if a small number of symptomatic, afebrile dengue patients seek care, our underreporting adjustments may be slightly conservative. We address this with a sensitivity analysis with different healthcare-seeking probabilities.

Our cost estimates only reflect dengue cases that sought medical care, consistent with data availability and reliability. We did not include costs associated with non-healthcare-seeking cases, such as informal care, lost productivity in mild cases, or over-the-counter medication use. While some studies estimate these costs, they represent a small proportion of the overall economic burden and are subject to considerable uncertainty. Therefore, while excluding non-medical cases in our study may slightly underestimate total societal economic burden, the impact is likely marginal.

Scenario analyses revealed that fatal cost estimates varied substantially depending on the valuation approach. Specifically, using a productivity-based approach resulted in lower estimates, whereas applying the VSL estimate from the U.S. Department of Transportation yielded higher estimates. Future research could investigate locally-derived estimates or mixed-method approaches that incorporate both methods.

The multiplier framework was calculated by comparing cases reported through multiple surveillance systems, healthcare facilities, municipalities, and timeframes; however, reporting rates vary over space and time. We addressed this by integrating setting-specific estimates—accounting for variations in healthcare-seeking and reporting by facility—into cross-setting underreporting estimates. Prior analyses have shown SEDSS trends reflect island-wide transmission patterns in Puerto Rico [34, 61]. Uncertainty in the final estimates is captured in the credible intervals, reflecting available data and populations sizes. Data limitations across sites and surveillance systems precluded estimation of additional subgroup-specific adjustment factors. Instead, we account for heterogeneity by generating overall adjustment factors that can be broadly applied over time across the island.

## Conclusions

This study highlights the substantial economic burden of dengue illness in Puerto Rico, with DMCs associated with hospitalization being the dominant drivers. By applying age- and hospitalization-specific multipliers to account for underreporting, we estimated the median aggregate annual cost of dengue illness to be 62.7 million USD (mean: 64.7 million) for the median incidence year over this time period (18 per capita USD) and 1.1 billion USD (mean: 1.1 billion) for an epidemic year (291 per capita USD). This substantial economic burden underscores the need for effective dengue prevention and control strategies. The significant increase in costs during epidemic years underscores the importance of robust surveillance systems and timely public health interventions. Cost-effectiveness analyses of different interventions can inform public health decision-making. By allocating resources towards interventions that target hospitalization reduction, such as improved case management, vaccination campaigns, or increased installation of indoor screens and air conditioning, policymakers can mitigate the economic burden of dengue in Puerto Rico [68]. This data provides valuable insights for healthcare providers and public health officials to develop effective strategies for managing and controlling dengue, ultimately protecting the health and well-being of the population.

## Supplementary Information

Supplementary file 1.

## Data Availability

Data cannot be shared publicly because data cannot be deidentified at the granular level of analyses performed. Data are available from the CDC management team (contact: dengue@cdc.gov) for researchers who meet the criteria for access to confidential data.

## Abbreviations

AM: Auxilio Mutuo Hospital
CEMI: Centro de Emergencia y Medicina Integrada
CMESL: Centro Médico Episcopal San Lucas
CrI: Credible interval
CSTE: Council of State and Territorial Epidemiologists
DENV: Dengue virus
DMC: Direct medical costs
DNMC: Direct non-medical costs
ELISA: Enzyme-linked immunosorbent assay
GDP: Gross domestic product
PADSS: Passive Arboviral Disease Surveillance System
PRDH: Puerto Rico Department of Health
RT-PCR: Reverse transcription-polymerase chain reaction
SEDSS: Sentinel Enhanced Dengue Surveillance System
VSL: Value of statistical life
