# Supplementary material for: Economic burden of dengue in Puerto Rico, 2010–2023
Source: Infect Dis Poverty. 2026 Jan 27;15:15. doi: 10.1186/s40249-026-01412-1 (PMC12837138; doi:10.1186/s40249-026-01412-1)
Supplement: Supplementary file 1 [file 40249_2026_1412_MOESM1_ESM.docx]

**Supplement**

**Supplementary Methods 1.** Questionnaire.

**Supplementary Methods 2.** Multiplier framework.

**Table S1.** Adjustment factor model parameters with their symbols, descriptions, uncertainty distributions, equations, and sources.

**Table S2.** Baseline characteristics of study participants.

**Table S3.** Detailed estimation of daily indirect costs of dengue.

**Table S4.** Estimated percentage of individuals attending (*p_attend_*), presenting (*p_present_*), and enrolling (*p_enroll_*) at the three sentinel surveillance sites (Auxilio Mutuo Hospital, San Lucas Episcopal Hospital, and Centro de Emergencia y Medicina Integrada), overall and for children and adults.

**Table S5.** Sensitivity analysis for the estimation of the average cost per case by care setting and type of cost.

**Table S6.** Sensitivity analysis for the aggregate annual cost of dengue with varying probabilities of healthcare seeking.

**Figure S1.** Reported and estimated outpatient, hospitalized, and fatal dengue cases for a median incidence year (2014) and an epidemic year (2010) by age group, Puerto Rico.

**Figure S2.** Cost components contributing to individual cost estimates by setting and age. The proportion of costs for each source (Centers for Disease Control and Prevention [CDC] lab tests, hospital financing, insurance schedules, or out-of-pocket expenses as reported in cost survey) is shown for hospitalized and outpatient children and adults.

**Figure S3.** Aggregate annual cost (in millions of US dollars) of projected dengue cases by setting and type of cost for a median incidence year (2014) and an epidemic year (2010), Puerto Rico. Costs are shown in millions (M) of 2023 USD.

**References**

**Supplementary Methods 1: Questionnaire**

CODE-PR – COst analysis of DEngue in Puerto Rico

**Participant Inclusion Criteria**

1. Participant name:

_________________________________ _________________________________

First last name Second last name

_________________________________ _________________________________

First name Middle name

1. Participant ID number: _______________
2. Case ID number: _______________
3. Participant phone number: _______________
4. Has the patient been diagnosed with dengue using a dengue laboratory test? (inclusion criterium) Yes No UK

*(If “Yes”, complete 5.1, 5.2 and 5.3)*

**5.1.** Laboratory confirmation of dengue was done through:

PCR

IgM Serology

PCR + IgM Serology

Other Specify _________________

**5.2.** Date of laboratory confirmation (date the first positive sample was collected)

m_____/d_____/y_____

**5.3.** Does the participant live in Puerto Rico?
Yes No

**Participant Informed Consent**

Interviewer name: _____________________________

Interview date: m_____/d_____/y_____

1. Who is answering the questionnaire?

Patient/ self

Mother/Father/Legal guardian/Caregiver

1. Patient’s date of birth: m_____/d_____/y_____
2. Sex:  Female  Male  NR
3. (Minor 14–20 years) Are you an emancipated minor?

No  Yes, I live independently  Yes, married  Yes, I have

from my parents children

1. (Adult 21+/emancipated minor) Adult or emancipated minor provides verbal consent:

I agree to respond to the survey

I agree to be contacted for this study if additional information is needed

1. (Minors 1–20 years) Parent or legal guardian provides verbal consent:

Survey

Contact for this study if additional information is needed

**12.** (5+ years) What is the highest level of education that you have obtained?

**Demographics**

| No school  Grades 1 to 5  Grades 6 to 8  Grades 9 to 11 | Completed grade 12/GED  Technical or associate’s degree  Bachelor’s degree | Professional degree  Post-graduate study  Special education  NR |
| --- | --- | --- |

**13.** (5+ years) Which of the following best describes your current employment status?

| Employed/full-time  Employed/part-time  Own business/self employed  Casual or informal work | Studying  Studying and working  Retired  Homemaker/ caretaker for family member | Health condition/ disability  Unemployed  Other  Specify _________________  NR |
| --- | --- | --- |

1. What is the approximately *total* annual household income? (include all sources of income in your household.)

| $__________ annual income | Prefer to respond in months:  $__________ per month | NR |
| --- | --- | --- |

1. How many people live in your house (sleeps 4 or more nights per week) including yourself? ______  NR

| **Sex:** | **Age (years):** | **Age (months):** *(for children under <1 year)* |
| --- | --- | --- |
| M / F / NR |  |  |
| M / F / NR |  |  |
| M / F / NR |  |  |
| M / F / NR |  |  |
| M / F / NR |  |  |
| M / F / NR |  |  |
| M / F / NR |  |  |
| M / F / NR |  |  |

M: male F: female NR: No response

1. Do you currently have medical insurance? (If “Yes”, ask 16.1.) Yes No UK NR

**16.1** Specify the type of plan: *(Mark all that apply.)*

| Reforma/ Plan Mi Salud  Medicare | Medicaid  Private  Tricare | Other: _________________  NR |
| --- | --- | --- |
| **Hospitalization costs** |  |  |

1. Did your dengue episode require you to stay overnight Yes No UK NR

in the health facility? *(If “****Yes****”, ask all the other questions in this section*

*If “****No****”, go to “Outpatient costs”)*

**17.1** In which health facility? **_________________________________________________________**

**17.2** How many nights did you spend in…?

17.2.1 Emergency room / Hospital (non-ICU) ________ days
17.2.2 ICU ________ days

≤ 30 minutes

30 – 60 minutes

≥60 minutes

- 1. How long did it take you to travel to the health facility (on the way there only)?

**17.4** Did you need to be taken to the health facility by ambulance? Yes No UK NR

17.4.1 How many times? **_____________________**

**17.5** When you had to stay overnight at a health facility, did you spend Yes No UK NR

any money on diagnosis tests, treatment, or other things like food or parking?

(If “Yes”, ask 17.6)

- 1. How much money did you spend on each of the following?
     1. Medical Consultation Fee / Copays **$**________
     2. Laboratory tests **$**________
     3. Medicines / prescriptions **$**________
     4. Food/drinks (meals) **$**________
     5. Transportation *(include Uber, taxis, buses, gas, tolls)* **$**________
     6. Parking **$**________
     7. Other (1) __________________________ **$**________
     8. Other (2) __________________________ **$**________

**Outpatient care costs**

1. Did you need to go to any of the following for the dengue episode?

(This includes all follow-up visits, excludes overnight stays)

**18.1** Emergency Room/Hospital Yes No

(If yes, answer 18.1.1 and 18.1.2)

18.1.1 How many times did you need to go to the hospital/emergency room?

(including all follow-up visits, excluding overnight stays)

________ times

18.1.2 How long did it take you to travel to the hospital/emergency room (on the way there only)?

≤ 30 minutes

30 – 60 minutes

≥60 minutes

**18.2** Clinic/Medical Office Yes No

(If yes, answer 18.2.1 and 18.2.2)

18.2.1 How many times did you need to go to the clinic/medical office?

(including all follow-up visits, excluding overnight stays)

________ times

18.2.2 How long did it take you to travel to the clinic/medical office (on the way there only)?

≤ 30 minutes

30 – 60 minutes

≥60 minutes

**18.3** Laboratory (to take blood samples, that are outside a clinic or hospital) Yes No

(If yes, answer 20.3.1 and 20.3.2)

18.3.1 How many times did you need to go to the laboratory? ________ times

18.3.2 How long did it take you to travel to the laboratory (on the way there only)?

≤ 30 minutes

30 – 60 minutes

≥60 minutes

If 18.1, 18.2, or 18.3 are “Yes” ask 18.4, 18.5

- 1. How much time did you spend at the health facility where you were diagnosed and/or treated (including waiting time)? (including all follow-up visits, excluding overnight stays)

Hours _________

Minutes ________

- 1. When you were sick with dengue, did you spend any money on diagnosis, treatment or other things like food, transport or parking? (including all follow-up visits, excluding overnight stays)
     1. Medical Consultation Fee / Copays **$**________
     2. Laboratory tests **$**________
     3. Medicines / prescriptions **$**________
     4. Food/drinks (meals) **$**________
     5. Transportation *(include Uber, taxis, buses, gas, tolls)* **$**________
     6. Parking **$**________
     7. Other (1) _____________________________ **$**________
     8. Other (2) _____________________________ **$**________
  2. How many days of work did you miss because of your dengue episode? (Only if patient works) __________ days *(if none, write 0)*
  3. How many days of class did you miss because of your dengue episode? (Only if patient studies) __________ days *(if none, write 0)*
  4. Did you have a caregiver to help with your illness or your family’s Yes No UK NR

daily needs (because of your dengue episode)? (If yes, ask 18.9)

- 1. Who was your caregiver?

Family member who lives with me

Family member who doesn’t live with me

Friend/neighbor

Paid caregiver

**18.10**  If paid caregiver, how much money did you spend on the caregiver?

**$**________

- 1. How many caregivers did you have?

___________

*(If more than 1, following questions will be answered for each caregiver)*

- 1. What does your caregiver normally do?

| Employed/full-time  Employed/part-time  Own business/self employed  Casual or informal work | Studying  Studying and working  Retired  Homemaker/ caretaker for family member | Health condition/ disability  Unemployed  Other  Specify _________________  NR |
| --- | --- | --- |

- 1. How many days was your caregiver looking after you without being able to perform other activities? ___________

**Supplementary Methods 2: Multiplier framework**

To accurately estimate the total number of acute febrile dengue cases seeking care in Puerto Rico, we developed a multiplier framework that accounts for underreporting in two different surveillance systems (Passive Arboviral Disease Surveillance System [PADSS] and Sentinel Enhanced Dengue Surveillance System [SEDSS]). This framework integrates site-specific data to provide a comprehensive estimate of dengue cases across the island. This model synthesizes data from three SEDSS sites and their respective municipalities: Auxilio Mutuo Hospital (AM) in San Juan (2018-2023), Centro Médico Episcopal San Lucas (CMESL) in Ponce (2012-2023), and Centro de Emergencia y Medicina Integrada (CEMI) in Ponce (2016-2023). We estimated these numbers separately for children (less than 18 years of age), adults (18 years and older), and overall for hospitalized (using data from AM and CMESL only) and outpatient (using data from all three sites) cases. In practice, SEDSS cases are also reported in PADSS, but here we differentiate between SEDSS cases and non-SEDSS cases in PADSS.

We developed a statistical framework to estimate the ratio of total acute febrile dengue cases receiving hospitalized or outpatient care relative to numbers of probable and confirmed cases reported in (non-SEDSS) PADSS. For one reported (non-SEDSS) PADSS case, the overall adjustment factor $\rho$ can be written as the ratio of SEDSS case incidence (adjusted for underreporting in SEDSS; $N_{adjusted.SEDSS}$) to (non-SEDSS) PADSS case incidence (adjusted for underreporting in non-SEDSS PADSS; $N_{adjusted.PADSS^{NS}}$; where superscript *NS* represents “non-SEDSS”):

$$\rho_{a}=\frac{N_{adjuste{d.SEDSS}_{a,s}}}{N_{adjusted.PADSS_{a,s}^{NS}}}$$

where subscript *a* represents age group (children ≤ 18 and adults > 18 years) and *s* represents the corresponding SEDSS site (AM, CMESL, and CEMI). We calculate these adjustment factors separately for hospitalized and outpatient cases, though not noted in the subscripts. All model input parameters with their distributions are listed in Table S1.

***Estimation of adjusted SEDSS dengue incidence***

For SEDSS, we adjusted the probable and confirmed dengue incidence for the probability of being reported within a catchment area ($p_{report.SEDSS};$the municipality where the SEDSS site is located), defined here as test sensitivity ($sensitivity$) and attendance ($p_{attend.SEDSS};$seeking care and enrolling) in SEDSS (Figure 1):

$p_{report.SEDSS_{a,s}}=p_{attend.SEDSS_{a,s}}*sensitivity$.

The adjusted incidence of dengue can be rewritten as the incidence of (confirmed and probable) dengue reported in SEDSS divided by the probability of being reported as a dengue case in SEDSS ($p_{report.SEDSS}$), which can be written as the product of the probability of attending a SEDSS facility for acute febrile dengue ($p_{attend.SEDSS}$) and the dengue test sensitivity ($sensitivity$) ($p_{report.SEDSS}=p_{attend.SEDSS}*sensitivity$),

$$N_{adjuste{dSEDSS}_{a,s}}=\frac{N_{reporte{d.SEDSS}_{a,s}}}{p_{report.SEDSS_{a,s}}}$$

We did not adjust for the specificity of the dengue diagnostic tests because their specificity is quite high [1, 2]. In other words, false positives are extremely rare and would not meaningfully inflate the number of reported cases. Furthermore, as noted later in the calculations, this part of the adjustment would cancel out even if it were to be included.

***Adjustment for the probability of attending a SEDSS facility***

The probability of attending a SEDSS facility ($p_{attend.SEDSS}$) represents the proportion of people with acute febrile illness who presented at a SEDSS facility within the catchment area, were eligible and enrolled. If people presented at a SEDSS facility and were eligible, 100% were enrolled and 100% were tested for dengue. The probability of attending a SEDSS facility is then the product of presenting and enrolling in that SEDSS facility ($p_{attend.SEDSS}=p_{present}*p_{enroll}$).

The probability of presenting at a SEDSS facility ($p_{present}$) was estimated differently for the three SEDSS sites due to data availability over the time periods when each site was open. For AM, this probability was estimated using non-SEDSS PADSS data from before 2018 (when the AM SEDSS site opened) to utilize more data and avoid the need to adjust for SEDSS enrollment. For CMESL, which opened in 2012, we used non-SEDSS PADSS data from 2012-2023, adjusting for the proportion enrolled in SEDSS (${1-p}_{enroll_{a,CMESL}}$). Since no (non-SEDSS) PADSS cases attended CEMI from Ponce, we calculated the probability of presenting at CEMI relative to CMESL using the enrollment-adjusted SEDSS cases from the two facilities from 2016-2023. Using reported data from SEDSS and (non-SEDSS) PADSS facilities to estimate the probability of presenting at a SEDSS site, we assumed that the probabilities follow a beta distribution,

$$p_{{present}_{a,AM}}\sim Beta(N_{reported.PADSS_{a,AM}^{NS}}, N_{reported.PADS{S.other}_{a,AM}^{NS}})$$

$$p_{{present}_{a,CMESL}}\sim Beta\left( \frac{N_{reported.PADSS_{a,CMESL}^{NS}}}{1-p_{enroll_{a,CMESL}}}, N_{reported.PADS{S.other}_{a,CMESL}^{NS}} \right)$$

$$p_{{present}_{a,CEMI}}\sim Beta\left( \frac{N_{reported.PADSS_{a,CMESL}^{NS}}}{1-p_{enroll_{a,CMESL}}}*\frac{\frac{N_{reported.PADSS_{a,CEMI}^{NS}}}{p_{enroll_{a,CEMI}}}}{\frac{N_{reported.PADSS_{a,CMESL\&CEMI}^{NS}}}{p_{enroll_{a,CMESL}}}}, N_{reported.PADS{S.other}_{a,CMESL}^{NS}} \right)$$

where $N_{reported.PADSS^{NS}}$ represents the reported number of (non-SEDSS) PADSS cases at the corresponding SEDSS facility among residents within the catchment area and $N_{reported.PADSS.other^{NS}}$ represents the reported number of (non-SEDSS) PADSS cases that were reported at other facilities from residents in the catchment area. We assumed a beta distribution for the probabilities of presenting and enrolling at SEDSS facilities, as this distribution is well-suited for modeling proportions and explicitly estimates uncertainty based on the observed data.We used the same method for estimating the probability of enrolling at a SEDSS facility for all three sites. Using SEDSS screening and eligibility data to estimate the probability of enrolling at a SEDSS site, we again assumed that the probability followed a beta distribution,

$$p_{enroll_{a,s}}\sim Beta(N_{{enroll.SEDSS}_{a,s}}, N_{{not.enroll.SEDSS}_{a,s}})$$

where $N_{{enroll.SEDSS}_{a,s}}$ and $N_{{not.enroll.SEDSS}_{a,s}}$represent the number of individuals at each SEDSS site who were eligible and not eligible to enroll based on the enrollment criteria, respectively.

***Adjustment for the probability of being reported at a (non-SEDSS) PADSS facility***

To be reported in (non-SEDSS) PADSS ($p_{report.PADSS}$), individuals must attend a PADSS facility ($p_{attend.PADSS}$) and then test positive for dengue ($sensitivity$):

$$p_{report.PADSS_{a,s}}=p_{attend.PADSS_{a,s}}*sensitivity.$$

Anyone who is seeking care for acute febrile dengue and does not attend a SEDSS facility could attend a PADSS facility within a SEDSS catchment area (i.e., they either present at a PADSS facility, or they present at a SEDSS facility but then do not enroll). In other words, the probability of attending (and being reported in) PADSS and not SEDSS, $p_{attend.PADSS_{a,s}}$, can be calculated as the complement of $p_{attend.SEDSS}$:

$$p_{attend.PADSS_{a,s}}=1-p_{attend.SEDSS_{a,s}}.$$

***Overall hospitalization and outpatient adjustment estimates***

The adjusted incidence of dengue cases in PADSS and SEDSS can then be rewritten:

$$N_{adjuste{d.SEDSS}_{a,s}}=\frac{N_{reporte{d.SEDSS}_{a,s}}}{p_{report.SEDSS_{a,s}}}=\frac{N_{reporte{d.SEDSS}_{a,s}}}{p_{attend.SEDSS_{a,s}}*sensitivity}$$

$$N_{adjusted.PADSS_{a,s}^{NS}}=\frac{N_{reported.PADSS_{a,s}^{NS}}}{p_{report.PADSS_{a,s}}}=\frac{N_{reported.PADSS_{a,s}^{NS}}}{p_{attend.PADSS_{a,s}}*sensitivity}$$

Substituting these estimates back into the first equation with cancellation of the sensitivity in each component yields:

$$\rho_{a}=\frac{N_{adjuste{d.SEDSS}_{a,s}}}{N_{adjusted.PADSS_{a,s}^{NS}}}=\frac{\frac{N_{reporte{dSEDSS}_{a,s}}}{p_{{attend.SEDSS}_{a,s}}}}{\frac{N_{reported.PADSS_{a,s}^{NS}}}{p_{attend.PADSS_{a,s}}}}$$

We can rearrange this equation to estimate the reported number of SEDSS cases in each site as a Poisson distribution,

$$N_{reporte{d.SEDSS}_{a,s}}\sim Poisson\left( \rho_{a}*p_{attend.SEDSS_{a,s}}*\frac{N_{reported.PADSS_{a,s}^{NS}}}{p_{attend.PADSS_{a,s}}} \right)$$

and applying a weakly informative Bayes-Laplace prior to the adjustment factor ($\rho$). In this way, the estimated reporting factor $\rho$ is comprised of the different probabilities of being reported in either surveillance system and the volume of cases reported in each surveillance system. This adjustment factor represents the estimated number of total acute febrile dengue patients seeking care for every confirmed dengue case reported in (non-SEDSS) PADSS. We integrate estimates from the three sites to arrive at one overall adjustment factor for the whole island. We leverage the relationship between the two surveillance systems in locations with SEDSS facilities to estimate overall underreporting of acute febrile illness dengue cases receiving hospitalized or outpatient care, regardless of location in Puerto Rico.

***Overall fatal case adjustment estimates***

For the underreporting adjustment for fatal cases, we employed a simpler approach. A previous study closely examined surveillance of fatal dengue-like acute febrile illnesses in Puerto Rico from 2010-2012[3]. From this study, we used the total number of fatal cases reported in PADSS out of the total number of laboratory-confirmed dengue fatal cases to calculate the multiplier.

***Model specification and diagnostics***

We fit the adjustment model to the SEDSS and (non-SEDSS) PADSS data using Markov chain Monte Carlo methods. To estimate the posterior distributions of the final multipliers, we collected 10,000 posterior samples following a burn-in period of 2,000 iterations across three chains prior to convergence. Convergence was assessed using the Gelman-Rubin diagnostic[4] for individual model parameters. All multiplier analyses were carried out using Stan version 2.21.0[5] in R version 4.1.1[6].

***Combining adjustment factors with cost estimates***

We obtained 10,000 posterior distributions of all adjustment factor parameters using Markov Chain Monte Carlo (MCMC) simulations in Stan. To combine these with the cost estimates, we drew 10,000 independent samples from a log-normal distribution using the mean and standard deviation of each cost parameter and verified that the mean and standard deviation were preserved in this distribution. We then combined the posterior distributions from the adjustment factors with the cost samples and computed the 95% credible intervals (CrIs) for each estimate using the 2.5^th^ and 97.5^th^ percentiles.

***Interpretation and use of adjustment factors***

The final multiplier $\rho$ represents the number of hospitalized or outpatient dengue cases for one reported (non-SEDSS) PADSS dengue case. The adjustment factors were intentionally created relative to non-SEDSS PADSS cases so they could be applied across Puerto Rico. Puerto Rico has PADSS sites across the island, but only has the three current SEDSS sites used in this study. In this way, we can apply the multipliers to all non-SEDSS PADSS cases in a year across the island to estimate all island-wide cases adjusted for underreporting, with the assumption that overall underreporting is similar in (non-SEDSS) PADSS sites and SEDSS sites. For example, suppose there were 100 reported non-SEDSS PADSS cases reported in a year. Multiplying that number (100) by the adjustment factor, 7.5 (95%CrI:6.2-9.0) for outpatient and 6.6 (95%CrI: 5.5-8.0) for hospitalized cases, yields total annual estimates of approximately 750 (95%CrI: 620-900) outpatient and 660 (95%CrI: 550-800) hospitalized dengue cases for that year.

Similarly, the multipliers for fatal cases are applied to reported non-SEDSS PADSS fatal dengue cases. Supposed there were 10 fatal dengue cases reported in (non-SEDSS) PADSS. Applying the overall fatal cases multiplier (1.1; 95%CrI: 1.1-1.3) yields a total of 11 (95%CrI: 11-13) fatal cases that year after adjusting for underreporting.

**Table S1. Adjustment factor model parameters with their symbols, descriptions, uncertainty distributions, equations, and sources.** SEDSS= Sentinel Enhanced Dengue Surveillance System; PADSS = Passive Arboviral Disease Surveillance System; AM = Auxilio Mutuo Hospital; CMESL= Centro Médico Episcopal San Lucas; CEMI=Centro de Emergencia y Medicina Integrada.

| **Symbol** | **Description** | **Uncertainty distribution, equation, or source** |
| --- | --- | --- |
| $\rho$ | Adjustment factor (i.e., multiplier or ratio of adjusted SEDSS to adjusted non-SEDSS PADSS incidence) | $\rho=\frac{N_{adjusted.SEDSS}}{N_{adjusted.PADSS^{NS}}}$  $\rho\sim Beta(1,1)$ |
| $N_{adjusted.SEDSS}$ | Adjusted incidence of dengue in SEDSS | $N_{adjusted.SEDSS}=\frac{N_{reported.SEDSS}}{p_{present}*p_{enroll}*sensitivity}=\frac{N_{reported.SEDSS}}{p_{attend.SEDSS}*sensitivity}=\frac{N_{reported.SEDSS}}{p_{report.SEDSS}}$ |
| $N_{adjusted.PADSS^{NS}}$ | Adjusted incidence of dengue in (non-SEDSS; NS) PADSS | $N_{adjusted.PADSS^{NS}}=\frac{N_{reported.PADSS^{NS}}}{p_{attend.PADSS}*sensitivity}$  $=\frac{N_{reported.PADSS^{NS}}}{p_{report.PADSS}}$ |
| $N_{reported.SEDSS}$ | Reported incidence of dengue in SEDSS | Observed directly from SEDSS |
| $p_{report.SEDSS}$ | Probability of being reported as a probable or confirmed dengue case in SEDSS | ${p_{report.SEDSS}=p_{attend.SEDSS}*sensitivity=p}_{present}*p_{enroll}*sensitivity$ |
| $p_{present}$ | Probability of presenting at a SEDSS facility | $p_{{present}_{AM}}\sim Beta(N_{reported.PADSS_{AM}^{NS}}, N_{reported.PADS{S.other}_{AM}^{NS}})$  $p_{{present}_{SLEH}}\sim Beta\left( \frac{N_{{reported.PAD{SS}_{CMESL}^{NS}}}}{1-p_{enroll_{CMESL}}}, N_{reported.PADSS.other_{CMESL}^{NS}} \right)$  $p_{{present}_{CEMI}}\sim Beta\left( \frac{N_{{reported.PAD{SS}_{CMESL}^{NS}}}}{1-p_{enroll_{CMESL}}}*\frac{\frac{N_{reported.PADSS_{CEMI}^{NS}}}{p_{enroll_{CEMI}}}}{\frac{N_{reported.PADSS_{CMESL\&CEMI}^{NS}}}{p_{enroll_{CMESL}}}},N_{reported.PADS{S.other}_{CMESL}^{NS}} \right)$ |
| $N_{reported.PADSS^{NS}}$ | Reported incidence of dengue at the corresponding SEDSS facility in PADSS within the catchment area | Observed directly from PADSS |
| $p_{report.SEDSS}$ | Probability of being reported as a probable or confirmed dengue case in (non-SEDSS) PADSS | $p_{report.PADSS}=p_{attend.PADSS}*sensitivity$ |
| $N_{reported.PADSS.other^{NS}}$ | Reported incidence of dengue *not* at the corresponding SEDSS facility in PADSS within the catchment area | Observed directly from PADSS |
| $p_{enroll}$ | The probability of being enrolled at a SEDSS facility (after presenting at the facility) | $p_{enroll}\sim Beta(N_{enroll.SEDSS}, N_{not.enroll.SEDSS})$ |
| $N_{enroll.SEDSS}$ | The number of individuals who presented at a SEDSS site and were eligible for enrollment (note: all eligible individuals are enrolled) | Observed directly from SEDSS |
| $N_{not.enroll.SEDSS}$ | The number of individuals who presented at a SEDSS site and were not eligible for enrollment | Observed directly from SEDSS |
| $sensitivity$ | Sensitivity of dengue diagnostic test | Not estimated (because it cancels out for SEDSS and PADSS) |
| $p_{attend.SEDSS}$ | Probability of attending (being reported at) a SEDSS facility for dengue | $p_{attend.SEDSS}=p_{enroll}*p_{present}$ |
| $p_{attend.PADSS}$ | Probability of attending (being reported at) a PADSS facility for dengue | $p_{attend.PADSS}=1-p_{attend.SEDSS}$ |

**Table S2**. Baseline characteristics of study participants

|  | Complete interviews (*n* = 101)  *n* (%) |
| --- | --- |
| Sex, female, n (%) | 50 (49.50) |
| Children (<18 years old), n (%) | 59 (58.42) |
| **Case classification**, n (%) | |
| - Confirmed dengue (positive PCR) | 97 (96.04) |
| - Probable dengue (clinical criteria + positive IgM) | 4 (3.96) |
| **Education grade**, n (%) (*n* = 96) | |
| - No school | 4 (4.17) |
| - Grade 1-5 | 24 (25.00) |
| - Grade 6-8 | 10 (10.42) |
| - Grade 9-11 | 19 (19.79) |
| - Grade 12 | 15 (15.63) |
| - Bachelor’s degree | 13 (13.54) |
| - Post-graduate | 4 (4.17) |
| - Professional degree | 3 (3.13) |
| - Technical-associated degree | 4 (4.17) |
| **Employment status**, n (%) (*n* = 96) | |
| - Studying* | 61 (63.54) |
| - Employed (full time) | 17 (17.71) |
| - Employed (partial time)* | 9 (9.38) |
| - Other employments | 6 (6.25) |
| - Retired /Disabled / Unemployed | 6 (6.25) |
| **Annual income ($),** Md (IQR) (*n* = 70) | 24,000 (12,000–36,000) |
| - <10,000 | 14 (20.00) |
| - 10,000-29,999 | 26 (37.14) |
| - 30,000-59,999 | 23 (32.86) |
| - ≥60,000 | 7 (10.00) |
| **Number of people living in the household**, n (%) (*n* = 106) | |
| - 1-2 | 21 (20.79) |
| - 3-4 | 60 (59.41) |
| - ≥ 5 (5-7) | 20 (19.80) |
| **Insurance**, n (%) (*n* = 100) | |
| - No insurance | 2 (2.00) |
| - Public | 54 (54.00) |
| - Private | 44 (44.00) |

* 3 participants studying and working.

**Table S3.** Detailed estimation of daily indirect costs of dengue. Both means with standard deviations and medians with 95% credible intervals are presented to provide a comprehensive summary of the results, as means are commonly used in economic analyses, while medians and credible intervals better reflect the skewed distribution of the data. All results are presented in 2023 US dollars (USD). SD = standard deviation; 95%CrI = 95% credible interval.

| **Setting and type of cost** | **Children** | | | | | **Adults** | | | | |
| --- | --- | --- | --- | --- | --- | --- | --- | --- | --- | --- |
|  | **Number of days** | | **Cost per day (USD)** | **Total cost (USD)** | | **Number of days** | | **Cost per day (USD)** | **Total cost (USD)** | |
|  | *Mean (SD)* | *Median (95%CrI)* |  | *Mean (SD)* | *Median (95%CrI)* | *Mean (SD)* | *Median (95%CrI)* |  | *Mean (SD)* | *Median (95%CrI)* |
| **Hospitalized** |  |  |  | **1446 (591)** | **1364**  **(527–2822)** |  |  |  | **1889 (1199)** | **1636**  **(314–4883)** |
| Patient | 16 (8) | 15 (4–33) | 51.30 | 812 (385) | 728  (302–1747) | 19 (15) | 15 (1–57) | 70.04 | 1335 (1054) | 1052  (256–4189) |
| Caregiver | 9 (6) | 8 (1–25) | 70.04 | 634 (448) | 520  (149–1797) | 8 (8) | 6 (0–29) | 70.04 | 554 (571) | 382  (68–2107) |
| **Outpatient** |  |  |  | **498 (238)** | **463**  **(144–1056)** |  |  |  | **794 (350)** | **749**  **(270–1647)** |
| Patient | 4 (3) | 3 (0–11) | 51.30 | 183 (148) | 143  (34–584) | 10 (4) | 9 (3–19) | 70.04 | 693 (295) | 632  (281–1394) |
| Caregiver | 5 (3) | 4 (0–11) | 70.04 | 315 (186) | 272  (93–805) | 1 (3) | <1 (<1–9) | 70.04 | 100 (189) | 46  (4–514) |
| **Fatal (total)** |  |  |  | **9,271,446 (2,446,250)** | **9,069,225**  **(5,094,937–14,672,800)** |  |  |  | **9,271,889 (2,446,250)** | **9,105,711**  **(5,046,180–14,744,352)** |
| Value of statistical life lost | - | - | - | 9,270,000 (2,446,250) | 8,938,783  (5,394,931–14,915,593) | - | - | - | 9,270,000 (2,446,250) | 8,938,783  (5,394,931–14,915,593) |

*Note: Cost per day for adult patients and all caregivers is to be estimated using Puerto Rico’s daily minimum wage (USD 70.04). Cost per day for pediatric patients is estimated using approach in Halasa (2012) of daily cost per pupil per day of schooling (USD 51.30).*

**Table S4.** Estimated percentage of individuals attending (*p_attend.SEDSS_*), presenting (*p_present_*), and enrolling (*p_enroll_*) at the three sentinel surveillance sites (Auxilio Mutuo Hospital, Centro Médico Episcopal San Lucas, and Centro de Emergencia y Medicina Integrada), overall and for children (<18 years) and adults (≥18 years). The median posterior probability estimates are presented with their 95% credible intervals (95% CrI).

|  |  | **Auxilio Mutuo Hospital** | **Centro Médico Episcopal San Lucas** | **Centro de Emergencia y Medicina Integrada** |
| --- | --- | --- | --- | --- |
| **Overall** | ***p_attend.SEDSS_*** | **2% (2**–**3%)** | **7% (6**–**8%)** | **2% (2** –**3%)** |
|  | *p_present_* | 20% (19–22%) | 29% (26–32%) | 4% (3–6%) |
|  | *p_enroll_* | 11% (9–13%) | 24% (22–27%) | 56% (49–62%) |
|  |  |  |  |  |
| **Children** | ***p_attend.SEDSS_*** | **5% (4**–**6%)** | **22% (18**–**27%)** | **3% (2**–**5%)** |
|  | *p_present_* | 17% (15–16%) | 39% (34–43%) | 4% (2–6%) |
|  | *p_enroll_* | 30% (23–38%) | 57% (51–63%) | 83% (71–91%) |
|  |  |  |  |  |
| **Adults** | ***p_attend.SEDSS_*** | **2% (1**–**2%)** | **3% (3**–**4%)** | **2% (1**–**3%)** |
|  | *p_present_* | 25% (22–28%) | 27% (23–31%) | 5% (3–7%) |
|  | *p_enroll_* | 8% (6–10%) | 12% (10–15%) | 47% (39–54%) |

**Table S5.** Sensitivity analysis for the estimation of the average and median cost per case by care setting and type of cost: scenario A (imputation of USD 0 for missing values in the interview), scenario B (hospitalization and ICU length of stay for fatal cases based on Tomashek KM. et al. *PLoS Negl Trop Dis*. 2016) [3], scenario C (approach for fatal cases based on economic value of years lost from premature death based on Halasa et al. *Am J Trop Med Hyg*. 2012.) [7]; scenario D (imputed value of statistical life hedonic wage method extrapolated to Puerto Rico from Mardones et al. *Latin American Research Review*. 2018); and scenario E (value of statistical life estimate from United States Department of Transportation). All results are presented in 2023 US dollars (USD). Both means with standard deviations and medians with 95% credible intervals are presented to provide a comprehensive summary of the results, as means are commonly used in economic analyses, while medians and credible intervals better reflect the skewed distribution of the data. Note that in some instances the median of the weighted average is larger than the median of the costs for children and adults due to the skewed distributions of costs and nonlinear nature of medians. SD = standard deviation; 95%CrI = 95% credible interval.

| **Setting and type of cost** | **Children** | | **Adults** | | **Weighted average** | |
| --- | --- | --- | --- | --- | --- | --- |
|  | *Mean USD (SD)* | *Median USD (95%CrI)* | *Mean USD (SD)* | *Median USD (95%CrI)* | *Mean USD (SD)* | *Median USD (95%CrI)* |
| **Hospitalized (total), Scenario A** | **5700**  **(2300)** | **5200**  **(2600–11,500)** | **7400**  **(3500)** | **6600**  **(2900–16,100)** | **7000**  **(3400)** | **6400**  **(3400–13,500)** |
| Direct medical | 3900  (2200) | 3400  (1200–9700) | 5300  (3200) | 4500  (1600–13,300) | 5000  (3000) | 4400  (2000–11,000) |
| Direct non-medical | 300  (300) | 200  (50–1000) | 200  (300) | 200  (30–1000) | 300  (300) | 200  (100–800) |
| Indirect | 1400  (700) | 1300  (500–3200) | 1800  (1500) | 1400  (300–5700) | 1700  (1400) | 1400  (500–4700) |
| **Outpatient (total), Scenario A** | **1300**  **(500)** | **1200**  **(600–2500)** | **1400**  **(400)** | **1400**  **(800–2400)** | **1400**  **(400)** | **1400**  **(900–2200)** |
| Direct medical | 700  (400) | 600  (200–1600) | 600  (200) | 500  (200–1100) | 600  (300) | 600  (300–1100) |
| Direct non-medical | 100  (200) | 100  (10–500) | 100  (100) | 100  (10–200) | 100  (100) | 100  (20–200) |
| Indirect | 500  (300) | 400  (200–1,200) | 800  (300) | 700  (300–1600) | 700  (300) | 700  (300–1400) |
| **Fatal (total), Scenario A** | **9,275,700**  **(2,446300)** | **8,972,800**  **(5,373,000–14,931,600)** | **9,277,400**  **(2,446,300)** | **8,998,600**  **(5,392,500–15,000,600)** | **9,277,200**  **(2,446,300)** | **9,031,100**  **(5,812,400–14,251,900)** |
| Direct medical | 3900  (2200) | 3500  (1200–9500) | 5300  (3200) | 4600  (1500–13,800) | 5100  (3100) | 4500  (1800–12,500) |
| Direct non-medical | 300  (300) | 200  (100–1000) | 200  (300) | 200  (30–1000) | 300  (300) | 200  (100–900) |
| Indirect | 9,271,400  (2,446,300) | 8,969,600  (5,366,300–14,928,900) | 9,271,800  (2,446300) | 8,995,000  (5,384,600–14,995,500) | 9,271,800  (2,446,300) | 9,025,600  (5,806,900–14,249,000) |
| **Fatal (total), Scenario B** | **9,280,100**  **(2,446,300)** | **8,971,600**  **(5,415,900–14,748,500)** | **9,280,100**  **(2,446,300)** | **8,980,100**  **(5,410,300–14,743,400)** | **9,280,100**  **(2,446,300)** | **9,008,800**  **(5,847,200–14,043,600)** |
| Direct medical | 8000  (3200) | $8,000  (6600–9800) | 8000  (3200) | 7300  (3300–16,400) | 8000  (3200) | 7400  (3900–15,300) |
| Direct non-medical | 300  (300) | 200  (100–1100) | 300  (300) | 200  (100–900) | 300  (300) | 200  (100–900) |
| Indirect | 9,271,800  (2,446,300) | 8,963,500  (5,406,600–14,740,200) | 9,271,800  (2,446,300) | 8,971,900  (5,401,800–14,735,700) | 9,271,800  (2,446,300) | 9,001,800  (5,840,700–14,036,700) |
| **Fatal (total), Scenario C** | **562,200**  **(29,700)** | **638,200**  **(635,900–644,600)** | **562,200**  **(29,700)** | **550,100**  **(546,900–559,200)** | **562,200**  **(29,700)** | **561,600**  **(558,800–569,600)** |
| Direct medical | 5200  (3100) | 3500  (1200–9900) | 5200  (3100) | 4600  (1600–13800) | 5200  (3100) | 4600  (1900–12600) |
| Direct non-medical | 300  (300) | 200  (100–1200) | 300  (300) | 200  (50–900) | 300  (300) | 200  (100–900) |
| Indirect | 556,700  (30,000) | 634,400  (634,400–634,400) | 556,700  (30,000) | 545,100  (545,100–545,100) | 556,700  (30,000) | 556,700  (556,700–556,700) |
| **Fatal (total), Scenario D** | **6,848,700**  **(2200)** | **6,848,200**  **(6,845,900–6,854,300)** | **6,850,000**  **(3200)** | **6,849,300**  **(6,846,200–6,858,400)** | **6,849,900**  **(3100)** | **6,849,200**  **(6,846,400–6,857,300)** |
| Direct medical | 5200  (3100) | 3500  (1200–9900) | 5200  (3100) | 4600  (1600–13,800) | 5200  (3100) | 4600  (1900–12,600) |
| Direct non-medical | 300  (300) | 200  (100–1200) | 300  (300) | 200  (50–900) | 300  (300) | 200  (100–900) |
| Indirect | 6,844,400  (0) | 6,844,400  (6,844,400–6,844,400) | 6,844,400  (0) | 6,844,400  (6,844,400–6,844,400) | 6,844,400  (0) | 6,844,400  (6,844,400–6,844,400) |
| **Fatal (total), Scenario E** | **13,204,300**  **(2200)** | **13,203,800**  **(13,201,500–13,209,700)** | **13,205,600**  **(3200)** | **13,204,900**  **(13,201,800–13,213,900)** | **13,205,500**  **(3100)** | **13,204,800**  **(13,202,100–13,212,700)** |
| Direct medical | 5200  (3100) | 3500  (1200–9900) | 5200  (3100) | 4600  (1600–13,800) | 5200  (3100) | 4600  (1900–12,600) |
| Direct non-medical | 300  (300) | 200  (100–1200) | 300  (300) | 200  (50–900) | 300  (300) | 200  (100–900) |
| Indirect | 13,200,000  (0) | 13,200,000  (13,200,000–13,200,000) | 13,200,000  (0) | 13,200,000  (13,200,000–13,200,000) | 13,200,000  (0) | 13,200,000  (13,200,000–13,200,000) |

**Table S6. Sensitivity analysis for the aggregate costs (in thousands of 2023 US dollars [USD]) of dengue illness with varying probabilities of healthcare seeking.** Aggregate costs in thousands of dollars are shown for a median incidence year, an epidemic year, and a long-term 8-year total (2010-2014, 2021-2023) in Puerto Rico. Both means with standard deviations and medians with 95% credible intervals are presented to provide a comprehensive summary of the results, as means are commonly used in economic analyses, while medians and credible intervals better reflect the skewed distribution of the data. The eight-year total represents long-term trends in Puerto Rico. The eight years in the long-term total include all years in the 2010–2023 period except for 2015–2020, which was marked by atypical dengue incidence in Puerto Rico likely due to the Zika epidemic and COVID-19 pandemic. SD = standard deviation; 95%CrI = 95% credible interval.

| **Change from base case probability of attending a SEDSS facility (*p_present_*)** | **Year** | **Aggregate costs (in thousands of 2023 USD)** | |
| --- | --- | --- | --- |
|  |  | *Mean (SD)* | *Median (95%CrI)* |
| **–20%** | *Median* | 75,800 (15,900) | 73,000 (52,900–113,300) |
|  | *Epidemic* | 1,296,600 (258,900) | 1,258,700 (913,200–1,909,900) |
|  | *8-year total* | 3,608,400 (763,300) | 3,490,100 (2,512,600–5,435,200) |
| **–10%** | *Median* | 72,500 (13,600) | 70,600 (52,000–104,400) |
|  | *Epidemic* | 1,235,600 (215,700) | 1,209,300 (894,800–1,734,600) |
|  | *8-year total* | 3,431,800 (631,300) | 3,346,900 (2,455,300–4,893,900) |
| **+10%** | *Median* | 58,300 (11,000) | 56,700 (41,900–83,900) |
|  | *Epidemic* | 1,026,500 (189,200) | 1,001,400 (729,900–1,462,400) |
|  | *8-year total* | 2,781,700 (538,400) | 2,703,000 (1,959,200–4,049,200) |
| **+20%** | *Median* | 57,200 (11,200) | 55,400 (40,800–83,400) |
|  | *Epidemic* | 987,400 (187,200) | 963,000 (697,100–1,418,600) |
|  | *8-year total* | 2,671,200 (533,500) | 2,593,000 (1,871,500–3,939,700) |

**Figure S1**. Reported and estimated outpatient, hospitalized, and fatal dengue cases for a median incidence year and an epidemic year by age group, Puerto Rico.


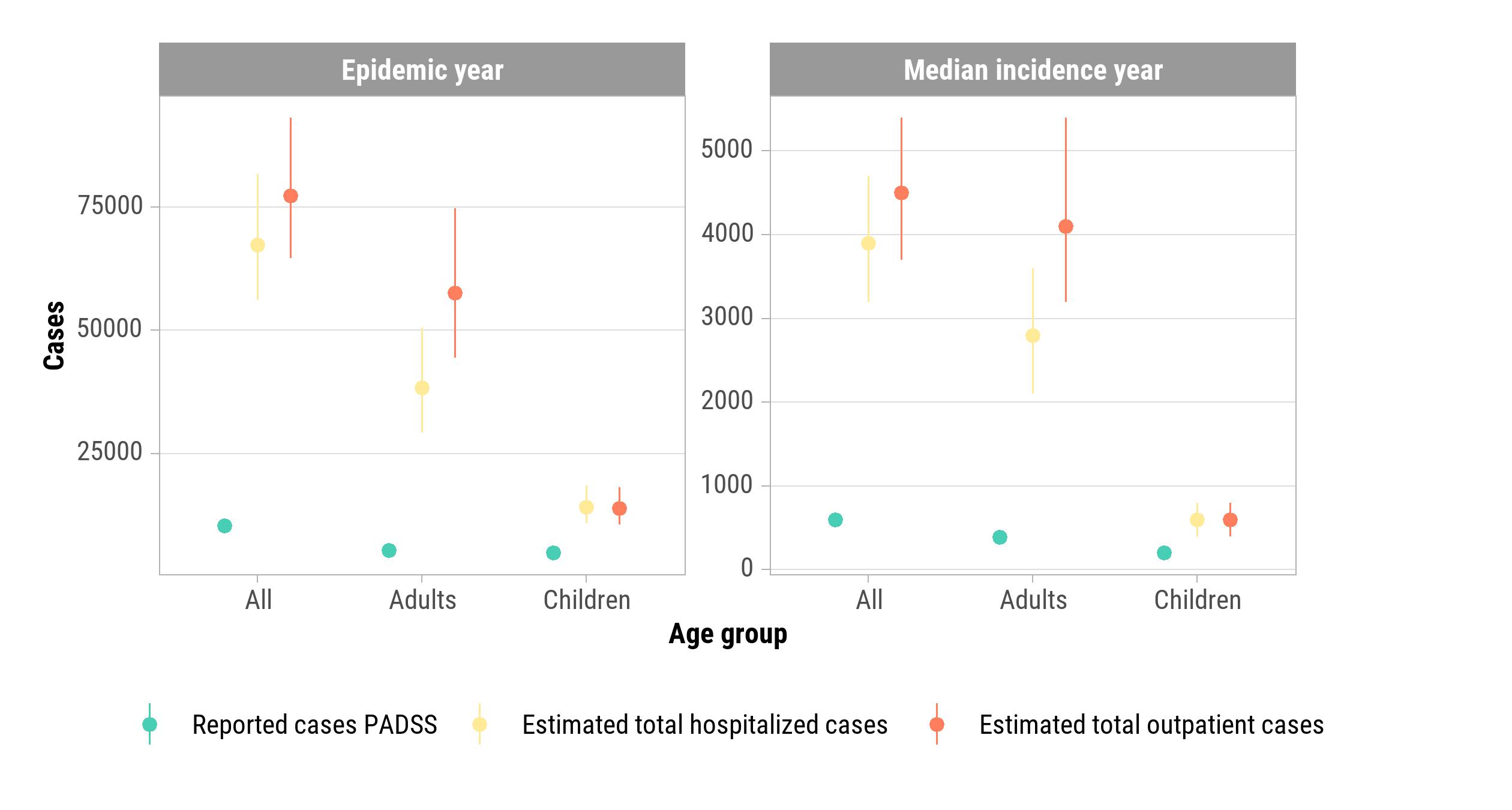


**Figure S2**. Cost components contributing to individual cost estimates by setting and age. The proportion of costs for each source (Centers for Disease Control and Prevention [CDC] lab tests, hospital financing, insurance schedules, or out-of-pocket expenses as reported in cost survey) is shown for hospitalized and outpatient children and adults.

**
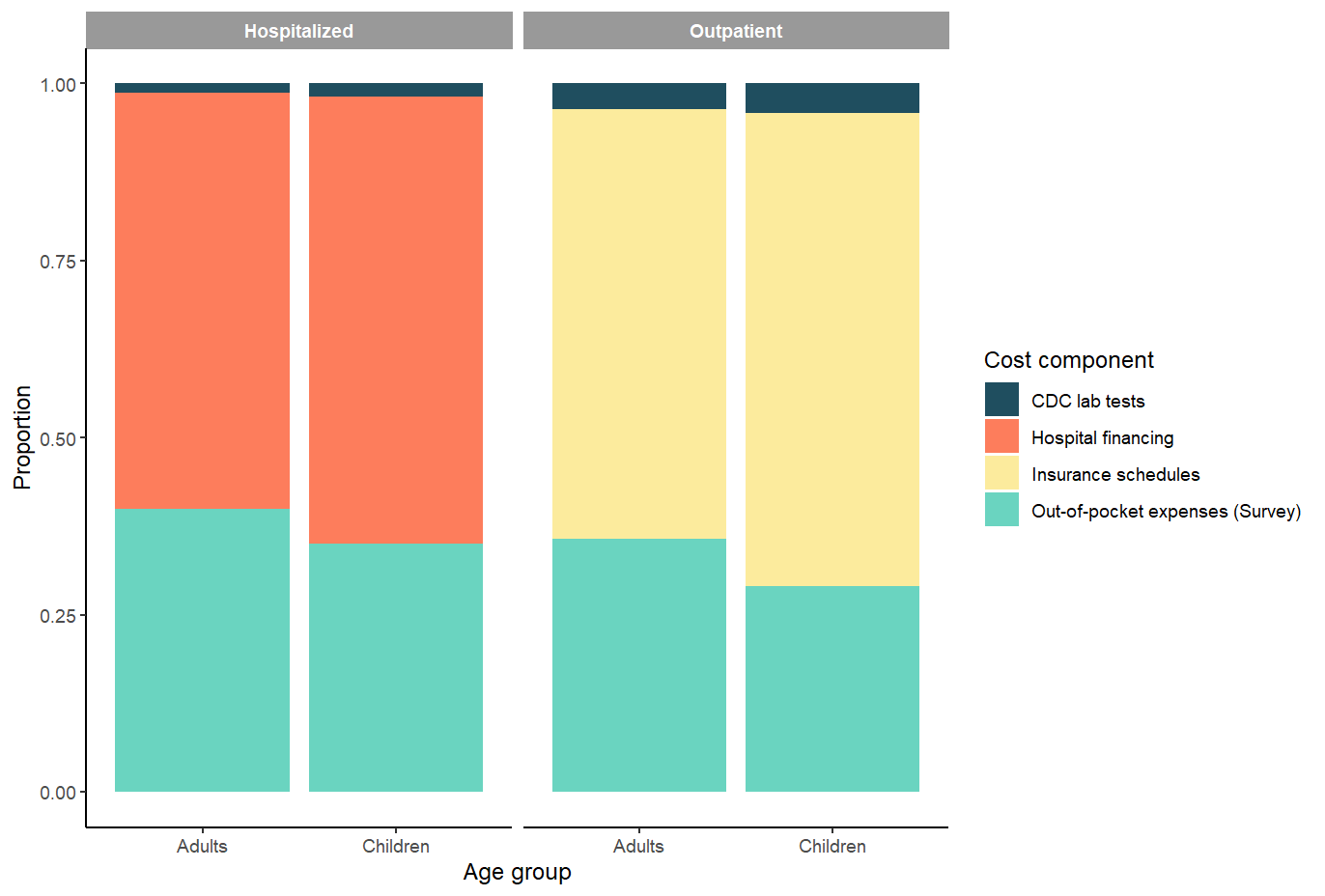
**

**Figure S3**. Aggregate annual cost (in millions) of projected dengue cases by setting and type of cost for a median incidence year and an epidemic year, Puerto Rico. Costs are shown in millions (M) of 2023 USD.


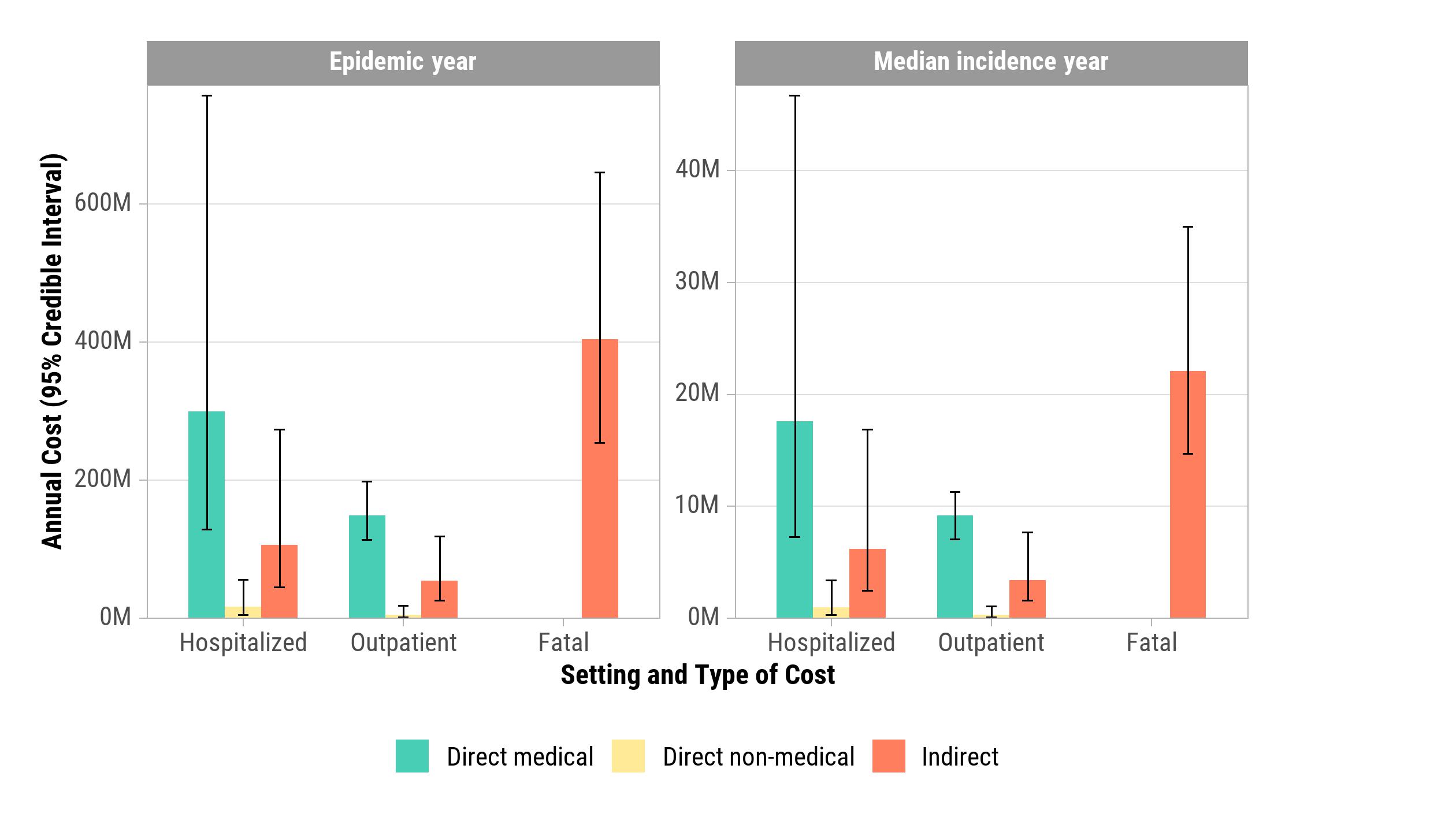


**References**

1. Ahmed NH, Broor S. **Comparison of NS1 antigen detection ELISA, real time RT-PCR and virus isolation for rapid diagnosis of dengue infection in acute phase**. *J Vector Borne Dis.* 2014;**51**(3):194-199.

2. Hunsperger EA, Yoksan S, Buchy P, Nguyen VC, Sekaran SD, Enria DA, *et al.***Evaluation of commercially available anti-dengue virus immunoglobulin M tests**. *Emerg Infect Dis.* 2009;**15**(3):436-440.

3. Tomashek KM, Rivera A, Torres-Velasquez B, Hunsperger EA, Munoz-Jordan JL, Sharp TM, *et al*. **Enhanced Surveillance for Fatal Dengue-Like Acute Febrile Illness in Puerto Rico, 2010-2012**. *PLoS Negl Trop Dis.* 2016;**10**(10):e0005025.

4. Gelman A, Rubin D. **Inference from iterative simulation using multiple sequences**. *Stat Sci.* 1992;**7**:457-472.

5. Stan Development Team. **Stan Modeling Language Users Guide and Reference Manual**. In*.*, 2.21.0 edn. 2024.

6. R Core Team. **R: A language and environment for statistical computing**. In*.* Vienna, Austria: R Foundation for Statistical Computing. 2021.

7. Halasa YA, Shepard DS, Zeng W. **Economic cost of dengue in Puerto Rico**. *Am J Trop Med Hyg.* 2012;**86**(5):745-752.
